# Design and Preclinical Evaluation of Two Prostate-Specific Membrane Antigen-Targeting Trifluoroborate Derivatives Suitable for One-Step Radiofluorination

**DOI:** 10.1021/acsomega.6c08595

**Published:** 2026-10-20

**Authors:** Andreas Paulus, Sofia Otaru, Risto Savela, Surachet Imlimthan, Tatsiana Auchynnikava, Dominika Novotná, Anisa Biti, Mirkka Sarparanta, Heidi Liljenbäck, Tatu Martinmäki, Alexander Popkov, Kim A. Bergström, Anne Roivainen, Anu J. Airaksinen

**Affiliations:** † Department of Chemistry Radiochemistry, 3835 University of Helsinki, Helsink FI-00014, Finland; ‡ Turku PET Centre, 176483 University of Turku and Turku University Hospital, Turku FI-20520, Finland; § Turku Center for Disease Modeling, University of Turku, Turku FI-20520, Finland; ∥ Institute of Organic Chemistry, 425573 Johannes Kepler University Linz, Linz A-4040, Austria; ⊥ Samo University, České Budĕjovice CZ-37001, Czech Republic; # HUS Diagnostic Center, Helsinki University Hospital and University of Helsinki, Helsinki FI-00290, Finland

## Abstract

Prostate cancer was the most frequently diagnosed cancer and the second leading cause of cancer-related death among men in the United States in 2025. Prostate-specific membrane antigen (PSMA)-targeted PET tracers have significantly improved the detection, visualization, and staging of prostate cancer. Recently, the novel prosthetic group [^18^F]­AmBF_3_-Tz was introduced, combining simple isotopic ^18^F-radiolabeling of alkylammoniomethyltrifluoroborates (AmBF_3_) with efficient bioorthogonal tetrazine-*trans*-cyclooctene (TCO) conjugation. This study evaluated the suitability of [^18^F]­AmBF_3_-Tz for radiolabeling PSMA-targeting urea derivatives and assessed its impact on PSMA-binding affinity in vitro and in vivo. Four precursors—TCO-PSMA, TCO-tranexamic acid-PSMA, AmBF_3_-PSMA, and AmBF_3_-tranexamic acid-PSMA—were synthesized and used for the preparation of [^18^F]­AmBF_3_-PSMA and [^18^F]­AmBF_3_-tranexamic acid-PSMA via one-step or two-step labeling strategies. Conjugation before radiolabeling gave radiochemical yields of 5.2–11.8%, whereas the two-step method gave 0.7–9.8%. Although these yields demonstrate feasibility, they are lower and more variable than the 33 ± 5% yield obtained for [^18^F]­AmBF_3_-Tz and would require substantial optimization before clinical translation. Overall, [^18^F]­AmBF_3_-PSMA and [^18^F]­AmBF_3_-tranexamic acid-PSMA demonstrated PSMA-specific tumor uptake and enabled the visualization of C4-2 xenografts. The tranexamic acid-containing tracer showed higher tumor-to-background ratios than [^18^F]­AmBF_3_-PSMA in this study, but the absolute tumor uptake was not significantly different between the two tracers. The moderate and variable radiochemical yields, microscale reaction sensitivity, and small animal group sizes indicate that this proof-of-concept platform warrants further optimization before its suitability for clinical translation can be assessed.

## Graphical abstract

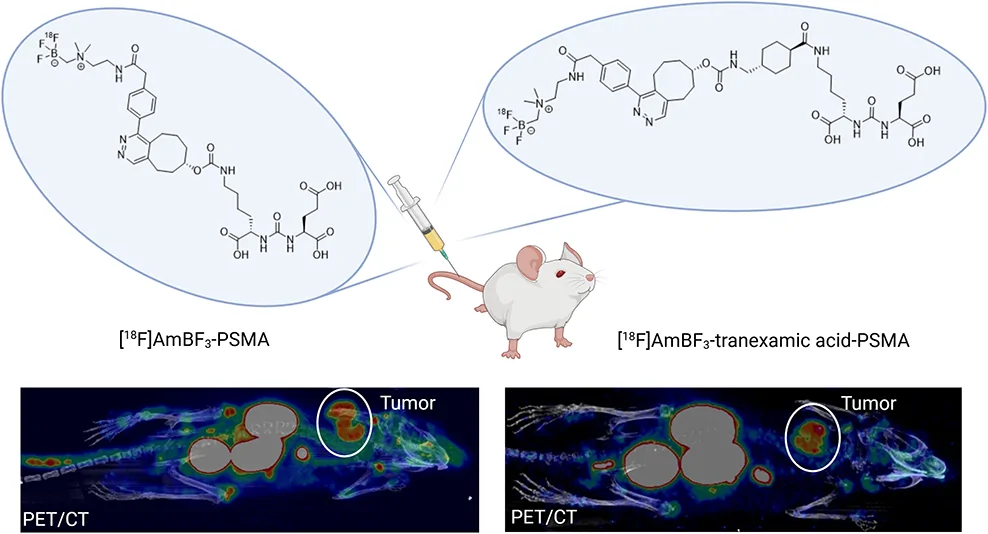

## 1 Introduction

Prostate cancer remains one of the most frequently diagnosed malignancies and is a leading cause of cancer-related mortality among men worldwide. Recent epidemiological projections indicate that both incidence and mortality are expected to increase further in the coming decades, emphasizing the need for improved diagnostic and therapeutic strategies.^1,2^

Prostate-specific membrane antigen (PSMA) has emerged as a highly attractive target for both imaging and therapy due to its strong overexpression in prostate cancer cells, particularly in advanced and metastatic disease, as well as its expression in tumor-associated neovasculature.^3–5^ Over the past two decades, extensive efforts have been devoted toward the development of PSMA-targeting agents, including antibodies,^6–10^ nanoparticles,^11–14^ and low-molecular-weight inhibitors.^4,11,15^ Among these, urea-based peptidomimetics have demonstrated particularly favorable pharmacokinetics and high binding affinity, leading to their widespread application in clinical imaging.^15–17^

Radiolabeled PSMA ligands such as [^68^Ga]­Ga-PSMA-11 are widely used for positron emission tomography (PET) and have significantly improved the detection of primary and metastatic lesions, particularly in patients with biochemical recurrence.^18,19^ However, the use of gallium-68 is associated with several limitations, including its relatively short physical half-life, high positron range, change of pharmacokinetics due to steric hindrance, introduction of possible charged centers in the molecule, and constraints in large-scale production due to generator-based production and distribution.^19^

In contrast, fluorine-18 offers several advantages, including a longer physical half-life (109.7 min), a lower positron energy and range resulting in improved spatial resolution, and the possibility for large-scale cyclotron production and centralized distribution.^16^ As a result, fluorine-18-labeled PSMA tracers such as [^18^F]­DCFPyL and [^18^F]­PSMA-1007 have gained increasing clinical importance and demonstrated excellent imaging performance.^16,20^ The growing clinical relevance of PSMA PET is further reflected in recent clinical guidelines, which increasingly support its use across PCa staging, restaging, and management.^18^

Despite these advances, many conventional fluorine-18 labeling strategies often require harsh, anhydrous reaction conditions, which can limit their applicability to sensitive biomolecules such as peptides and peptidomimetics.^21^ To overcome these limitations, alternative radiochemistry approaches have been developed (e.g., [^18^F]­DCFpyL). In particular, organotrifluoroborate-based strategies, especially zwitterionic alkylammoniomethyltrifluoroborates (AmBF_3_), enable efficient radiolabeling via isotopic exchange under mild, aqueous conditions while maintaining high radiochemical yields (RCYs).^21–23^

In parallel, bioorthogonal chemistry has gained increasing importance in radiopharmaceutical development. Among available bioorthogonal reactions, the inverse-electron-demand Diels–Alder (IEDDA) reaction between tetrazines (Tz) and *trans*-cyclooctene (TCO) is especially attractive due to its rapid reaction kinetics, high selectivity, and compatibility with biological systems.^24^ Recent developments combining AmBF_3_ chemistry with tetrazine-based prosthetic groups provide a promising strategy for efficient and mild radiolabeling workflows.^23^

Organotrifluoroborate-based radioprosthetic groups have further expanded the toolbox for one-step fluorine-18-labeling strategies via isotopic exchange, enabling simplified radiochemistry under mild conditions while maintaining high molar activity and suitable *in vivo* performance. In particular, the development of imidazolium-methylene trifluoroborate (ImMBF_3_) demonstrated that tuning the electronic properties of the borate scaffold can significantly improve radiochemical conversion, molar activity, and overall radiosynthetic efficiency, without compromising PSMA-targeting capability or tumor uptake in vivo.^25^ These findings highlight how subtle structural modifications of radioprosthetic groups can directly influence both labeling efficiency and *in vivo* behavior.

Beyond single-modality PET imaging, the integration of optical and nuclear imaging has emerged as a powerful strategy for image-guided oncology. Dual-mode PSMA-targeting tracers incorporating organotrifluoroborates, fluorescent dyes (e.g., fluorescein (FAM) and fluorescein isothiocyanate (FITC)), and clinically validated PSMA-binding scaffolds such as PSMA-617 and PSMA-1007 enable simultaneous PET signal generation and fluorescence visualization and therefore allow both diagnostic imaging and intraoperative guidance within a single molecular platform.^26^

The translational potential of such dual-modality systems has been further confirmed in clinical settings. First-in-human evaluation of the PSMA-targeted agent [^18^F]­BF_3_-Cy3-ACUPA demonstrated safety, favorable biodistribution, and effective tumor visualization in patients with PSMA-positive prostate cancer. Importantly, the tracer enabled not only preoperative PET/computed tomography (CT) imaging but also intraoperative fluorescence-guided visualization of tumor tissue up to 24 h postinjection, thereby bridging diagnostic imaging and surgical guidance within a single administration.^27^

Complementing these developments, PSMA-targeting probes based on trifluoroborate prosthetic groups have been shown to consistently achieve high tumor uptake, efficient one-step radiolabeling, and favorable tumor-to-background ratios in PSMA-expressing LNCaP xenograft models. Systematic variation of linker structures and prosthetic group chemistry significantly influenced pharmacokinetics, clearance pathways, and image contrast, with several optimized constructs comparable to clinically established tracers such as [^18^F]­DCFPyL.^28^

Together, these findings reinforce the versatility of organotrifluoroborate chemistry for designing next-generation PSMA-targeted imaging agents. However, despite the promising characteristics of the recently introduced [^18^F]­AmBF_3_-Tz prosthetic group, its applicability to low-molecular-weight PSMA-targeting peptidomimetics has not yet been systematically investigated.

In this work, we investigate the suitability of the recently developed [^18^F]­AmBF_3_-Tz prosthetic group for the development of fluorine-18 radiolabeled PSMA-targeting tracers. Two Glu-urea-Lys derivatives containing different spacer structures between the PSMA-binding motif and the AmBF_3_-Tz moiety were synthesized via two synthetic pathways and evaluated. Two radiolabeling approaches were compared: (i) radiolabeling of AmBF_3_-Tz (**1**) prior to conjugation with the TCO-functionalized PSMA precursor and (ii) radiolabeling following Tz-TCO conjugation (Figure 1). In addition, the resulting PSMA-targeting tracers were evaluated for uptake in PSMA-expressing human prostate cancer C4-2 cells *in vitro*. Their *in vivo* pharmacokinetics and tumor-targeting properties were further assessed by PET imaging and *ex vivo* biodistribution studies in nude mice bearing ectopic C4-2 xenografts.

**Figure 1:**
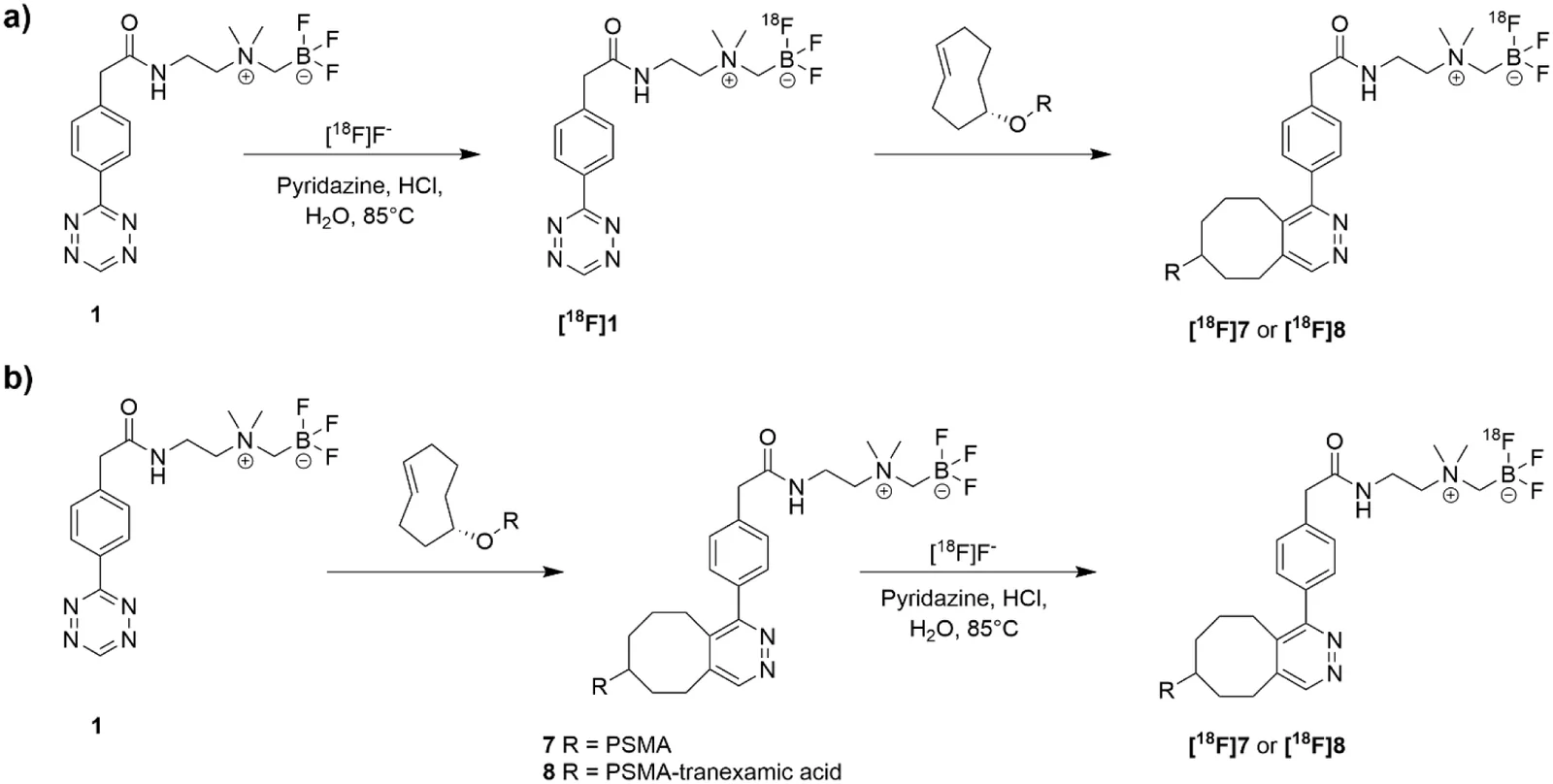
Schematic procedure of synthesis options for ^18^F-labeled PSMA derivatives in this study: a) Radiolabeling of **1** and subsequent conjugation to the TCO-functionalized PSMA derivative (R) via IEDDA reaction. b) Conjugation of **1** to the TCO-functionalized PSMA derivative (R) via IEDDA reaction and radiolabeling of the conjugate.

## 2 Results

### 2.1 Synthesis of AmBF_3_-Tz (**1**)

Compound **1** was synthesized as reported before by a four-step procedure.^23^ As all reaction steps yielded almost quantitative conversion to the product, HPLC purification was only necessary after the final step, resulting in an overall yield of 39.4%, n = 6. Characterization was done by LC-MS and NMR and revealed the correct identity of compound **1**.

### 2.2 Synthesis of PSMA-TCO (**2**) and PSMA-Tranexamic Acid-TCO (**6**)

PSMA derivative **2** was synthesized by a one-step reaction starting from commercially available PSMA-amine, which was further conjugated with a TCO-NHS ester at 71% yield after HPLC purification (Figure 2). Characterization was done by NMR and LC-MS (Supplementary Figures S1, S2, and S5). Derivative **6** was synthesized by a four-step reaction starting from t-butyl-Glu-Urea-Lys and FMOC-protected tranexamic acid (Figure 2). After conjugation, both protecting groups were cleaved off prior to conjugation with TCO-NHS. Additionally, **6** was synthesized with an alternative route (Figure 3). The final product **6** was purified by HPLC with an overall yield of 41%. Characterization was done by NMR and LC-MS (Supplementary Figures S3, S4, and S5).

**Figure 2:**
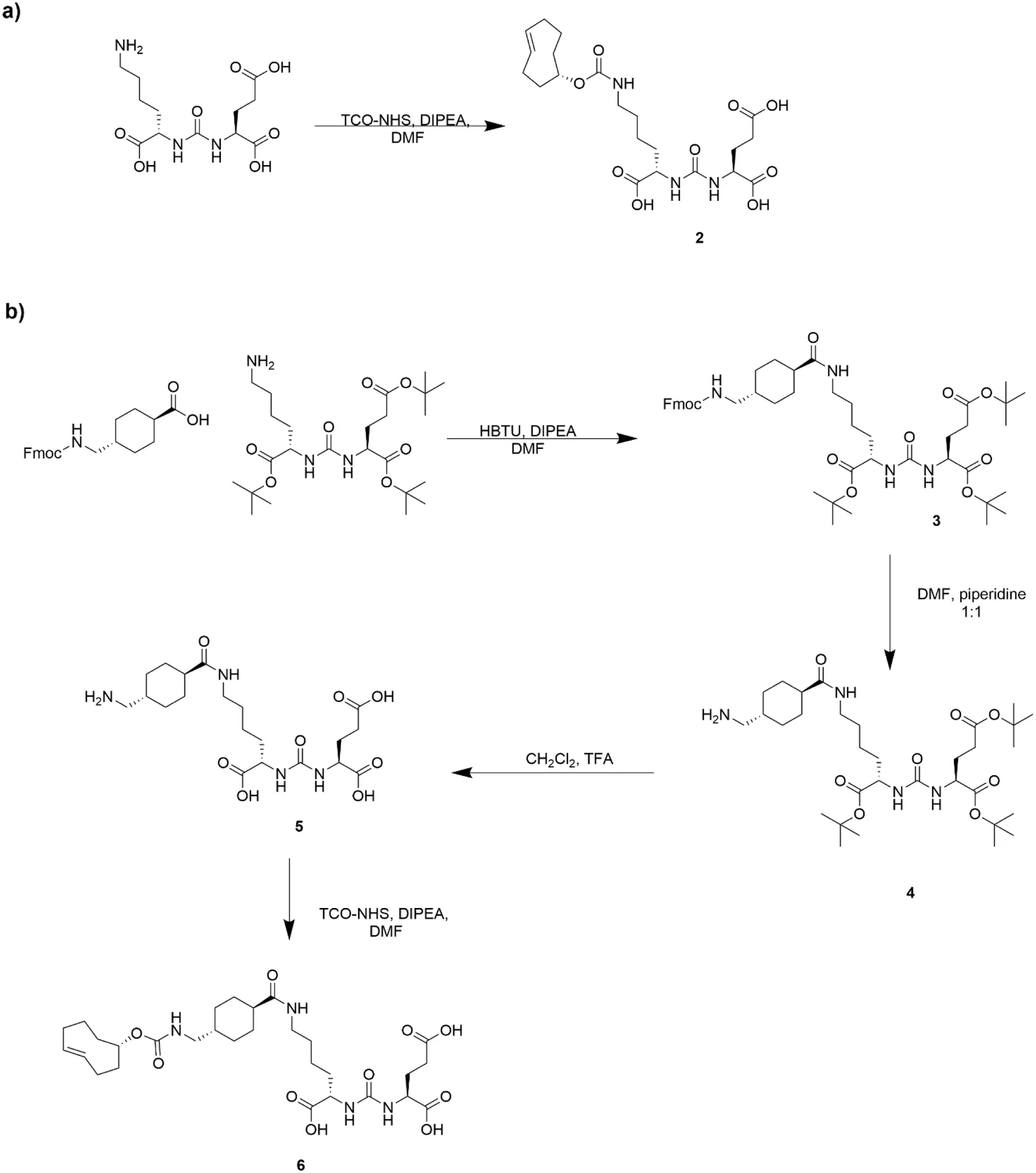
Synthesis pathways of a) TCO-functionalized PSMA (**2**) and b) TCO-functionalized tranexamic acid-PSMA (**6**).

**Figure 3:**
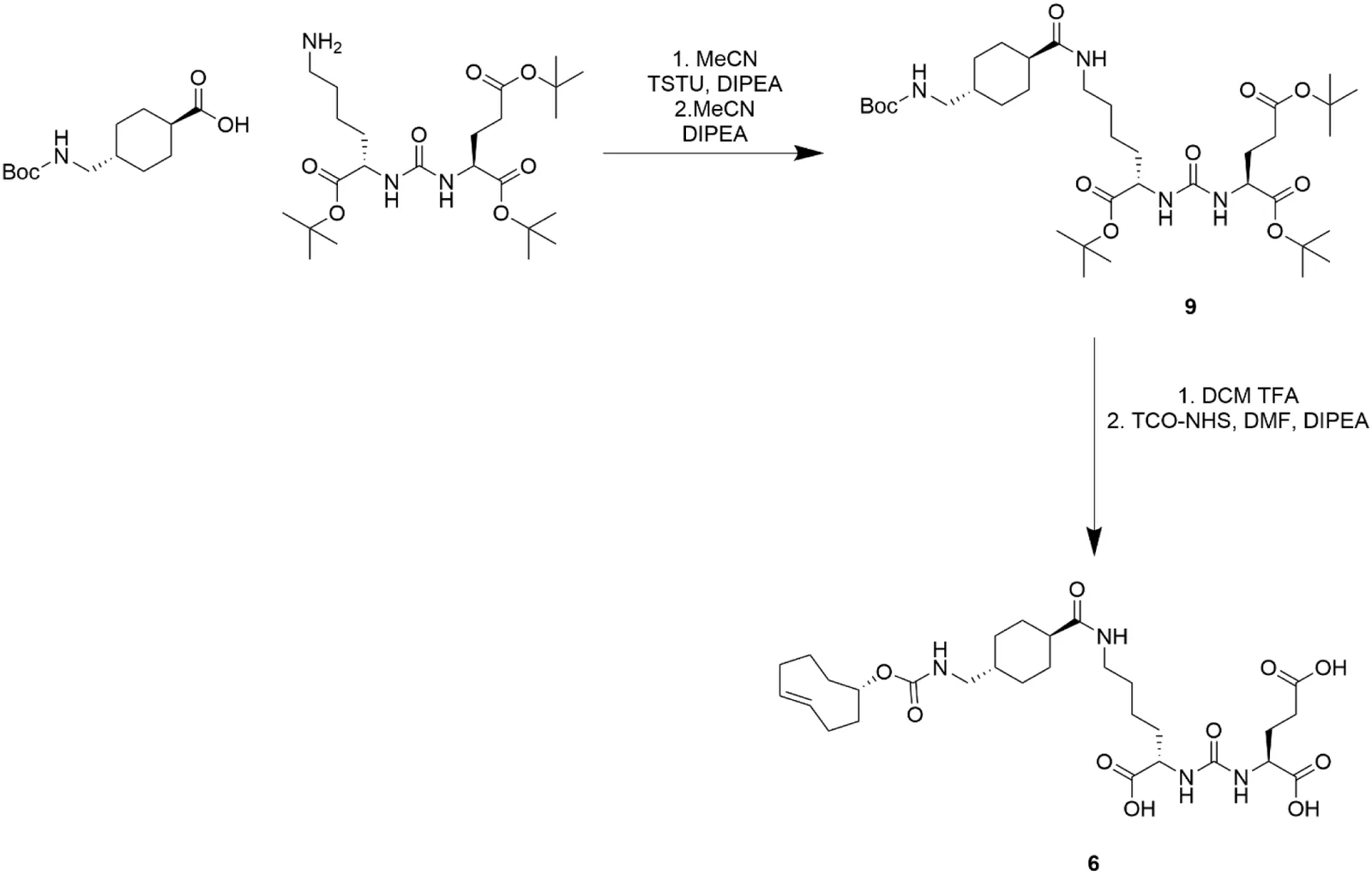
Alternative synthesis pathway to TCO-functionalized tranexamic acid-PSMA (**6**).

### 2.3 IEDDA Conjugation of AmBF_3_-Tz (**1**) or [^18^F]­AmBF_3_-Tz (**[^18^F]­1**) with PSMA-TCO (**2**) and PSMA-Tranexamic Acid-TCO (**6**)

**1** or [^**18**^ **F]­1** was reacted with TCO-functionalized PSMA derivative **2** or **6** in the presence of pyridazine HCl buffer (pH 2) in equimolar amounts at 60 °C. In a previous publication, we reported the importance of temperature and solvent composition for the total aromatization of the TCO-tetrazine cycloaddition product.^29^ It was observed that in the case of PSMA analogs, pH was also a critical parameter, as a more basic pH resulted in the formation of more side products. After HPLC purification, the IEDDA reaction resulted in a 48–49% yield of the product which was analyzed by LC-MS (Supplementary Figures S5–S7).

### 2.4 Radiolabeling of AmBF_3_-Tz (**1**), AmBF_3_-PSMA (**7**), and AmBF_3_-Tranexamic Acid-PSMA (**8**)

**1**, **7**, and **8** were radiolabeled with the same experimental procedure where [^18^F]­fluoride was concentrated in an aqueous medium and the radiolabeling of the precursor was done at 85 °C and pH 2. Fluorine-18 incorporation yields for [^**18**^ **F]­1** were 33 ± 5%, n = 6. [^**18**^ **F]­7** and [^**18**^ **F]­8** were purified resulting in an radiochemical purity (RCP) of >95% analyzed by radio-HPLC and radio-TLC (Supplementary Figure S14). The RCY for [^**18**^ **F]­7** and [^**18**^ **F]­8** ranged from 5.2 to 11.8%. During the optimization process of the radiolabeling, the following observations were made: the labeling reaction is dependent on the molar amount (100 nmol was the limit, and lower molar amounts resulted in decreased RCY), and the labeling should be done in the microliter scale (<30 μL final reaction volume). Consequently, due to the unavailability of a microfluidic system, the labeling was poorly reproducible and needed good knowledge and training to carry out successfully, as the reaction volumes were extremely small (15–30 μL) and even the slightest of deviations in the reaction conditions led to a significant decrease in RCY.^23^ The shelf life and plasma stability have been reported for [^**18**^ **F]­1**,^23^ and no deviations for [^**18**^ **F]­7** and [^**18**^ **F]­8** from the original data could be observed here, as both compounds showed stability >95% after 4 or 6 h, respectively. The log*D* values were −2.74 ± 0.09 for [^**18**^ **F]­7** and −1.80 ± 0.36 for [^**18**^ **F]­8**. Radiolabeling of **1** was carried out as previously reported with comparable yields (nonisolated RCY of 33 ± 5%, n = 6).^23^

### 2.5 *In Vitro* Cell Uptake

Cellular uptake of [^**18**^ **F]­7** and [^**18**^ **F]­8** in PSMA-positive C4-2 cells was studied over 2 h, and the cellular uptake was challenged by 2-PMPA as a blocking agent. For [^**18**^ **F]­7**, the highest cellular uptake was found at 30 min (2.44 ± 0.14%), whereas at later time points, the uptake slightly decreased (2.05 ± 0.28% for 60 min and 1.24 ± 0.47% for 120 min). Uptake at all time points was significantly inhibited (n = 3, *p* < 0.01) in blocking conditions at a constant level of 0.53 ± 0.13%. The ratio between internalized and overall cell-associated uptake increased from 21 ± 3.4% at 30 min to 34 ± 5.7% at 120 min (Figure 4).

**Figure 4:**
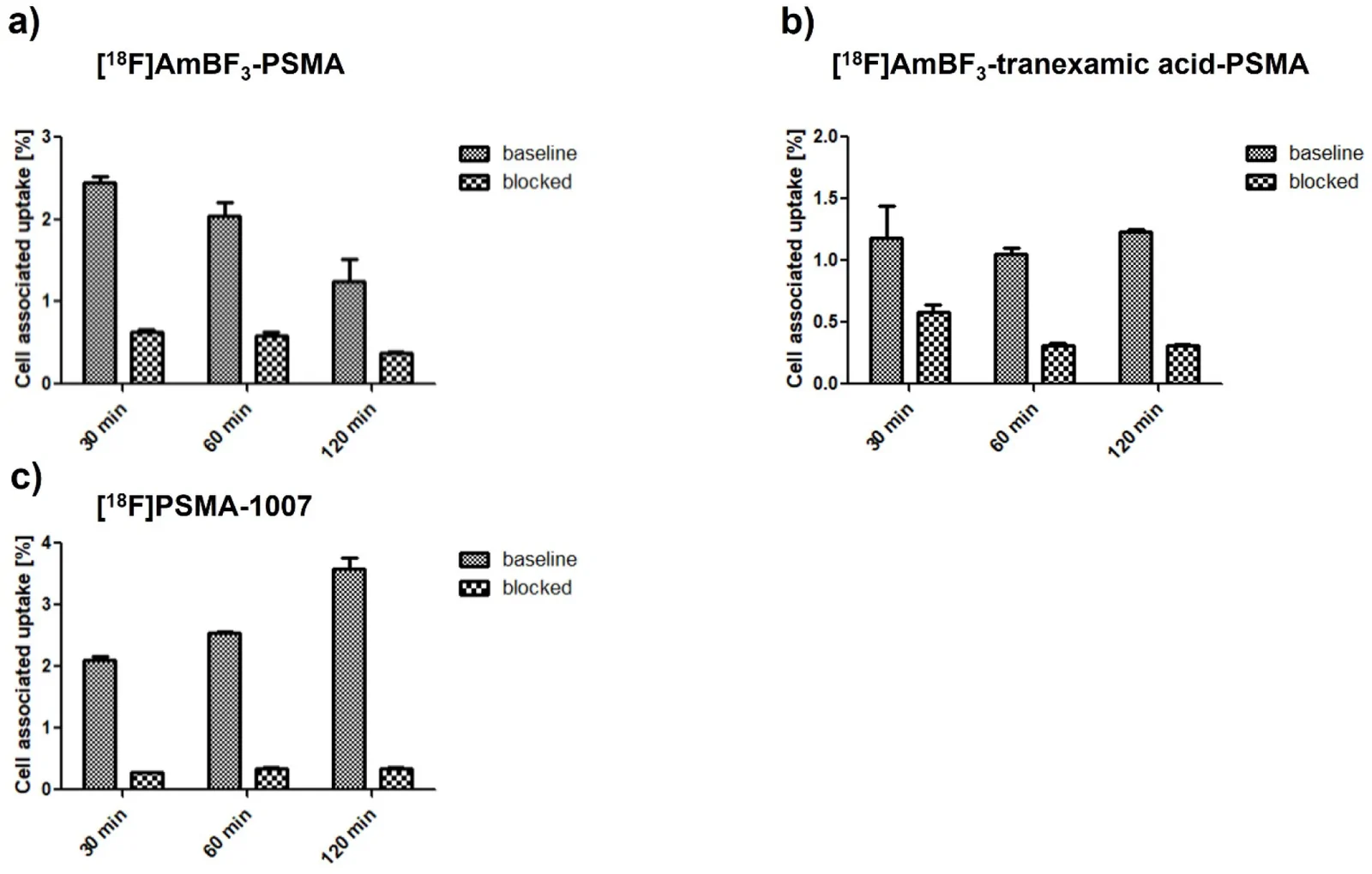
Cell-associated uptake of a) [^18^F]­AmBF_3_-PSMA (**[^18^F]­7**), b) [^18^F]­AmBF_3_-tranexamic acid-PSMA (**[^18^F]­8**), and c) [^18^F]­PSMA-1007 in C4-2 cells (6 × 10^5^ cells/well); 1 mL of a 250 nM tracer solution was incubated for 30, 60, and 120 min. Uptake was challenged by coincubation with a 400 μM 2-PMPA solution.

For [^**18**^ **F]­8**, the highest cell-associated uptake was found at 120 min (1.23 ± 0.03%), whereas at earlier time points, the uptake was comparable (1.18 ± 0.45% for 30 min and 1.05 ± 0.09% for 60 min). Uptake at all time points was significantly decreased (n = 3, *p* < 0.01) for blocking conditions to a constant level of 0.41 ± 0.14%. The ratio of internalized vs overall cell-associated uptake increased from 19 ± 3.9% at 30 min to 41 ± 4.6% at 120 min (Figure 4).

A comparison with [^18^F]­PSMA-1007 (2.11 ± 0.10% for 30 min, 2.55 ± 0.04% for 60 min, 3.59 ± 0.29% for 120 min) showed significantly lower uptake for [^**18**^ **F]­8** (n = 3, *p* > 0.05), whereas no significant difference can be reported for [^**18**^ **F]­7** (n = 6, *p* < 0.05). Internalization ratios for [^**18**^ **F]­7** and [^**18**^ **F]­8** were found to be lower compared to [^18^F]­PSMA-1007 but only [^**18**^ **F]­7** showed a significant difference (n = 6, *p* < 0.05) (Figure 4). Both compounds [^**18**^ **F]­7** and [^**18**^ **F]­8** showed specific uptake in C4-2 cells.

### 2.6 *Ex Vivo* Biodistribution

[^**18**^ **F]­7** or [^**18**^ **F]­8** (1 nmol, 100 μL in sterile 1 × PBS) was injected into awake C4-2 tumor-bearing SCID mice, and tumor uptake was challenged by preinjection of 2-PMPA (40 nmol, 100 μL) 30 min prior to the radiotracer administration. Animals were sacrificed by CO_2_ asphyxiation followed by cervical dislocation after 60 min, organs were collected, and radioactivity distribution was determined with a γ-counter and calculated as the percentage of injected dose per gram of tissue (%ID/g) (Figure 5). [^**18**^ **F]­7** showed the highest uptake in kidneys (128.7 ± 35.0%ID/g), as kidneys express PSMA endogenously,^3,30^ and this is also the predominant way of excretion of PSMA compounds reported in the literature.^31^ Increased tracer uptake was also observed in the gallbladder (30.8 ± 4.6%ID/g), spleen (21.2 ± 12.1%ID/g), and small intestine (11.5 ± 4.2%ID/g). Tumor accumulation was found to be 7.5 ± 0.7%ID/g and was significantly reduced by pretreatment with 2-PMPA to 1.2 ± 0.2%ID/g (n = 6, *p* < 0.01, Figure 5 and Supplementary Figure S15).

**Figure 5:**
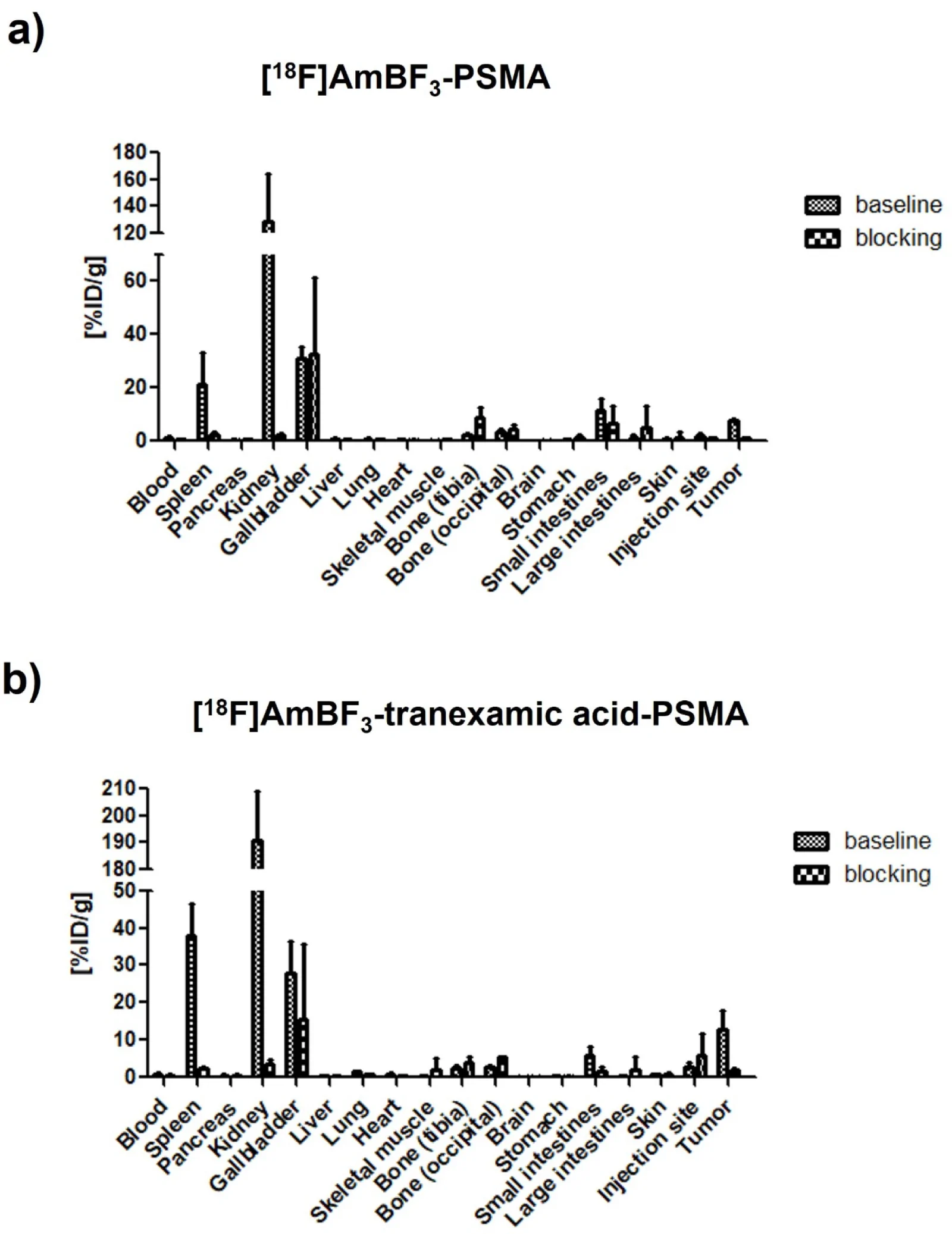
Biodistribution results of a) [^18^F]­AmBF_3_-PSMA [**^18^F]­7** and b) [^18^F]­AmBF_3_-tranexamic acid-PSMA [**^18^F]­8** after 60 min (100 μL injected, 0.01 mM solution in PBS) in awake animals. Blocking was performed by injection of 2-PMPA (100 μL, 0.4 mM) 30 min prior to the tracer injection.

Uptake of **[^18^F]­8** in the tumor was 12.9 ± 4.8%ID/g and was significantly reduced by the administration of 2-PMPA to 1.2 ± 0.2%ID/g (n = 6, p = 0.05).

Uptake in organs (kidneys and spleen) expressing PSMA endogenously was also reduced by blocking with 2-PMPA for **[^18^F]­7** or **[^18^F]­8** (Figure 5 and Supplementary Figure S15).

Both tracers exhibited some bone uptake (**[^18^F]­7**: 2.2 ± 0.8%ID/g; **[^18^F]­8**: 2.4 ± 0.7%ID/g), which increased during blocking conditions (**[^18^F]­7**: 8.5 ± 4.3%ID/g; **[^18^F]­8**: 4.1 ± 1.3%ID/g). This counterintuitive increase was not anticipated with PSMA blocking. A plausible explanation is that blocking altered PSMA-mediated renal handling and overall clearance, thereby increasing the fraction of circulating activity available for nonspecific bone-associated accumulation; in vivo defluorination cannot be excluded. Because bone radioactivity was not chemically characterized and blood/plasma kinetics were not measured in parallel, this interpretation remains tentative and warrants dedicated metabolite and time-course studies.

### 2.7 PET/CT Imaging

Results of the *ex vivo* biodistribution are supported by the PET/CT images. The radiotracer, **[^18^F]­7** or **[^18^F]­8**, was administered as a 0.01 mM solution in 1 × PBS into anesthetized animals (1–2% isoflurane) before the dynamic PET scan was carried out for 90 min. Animals with blocking conditions were injected 30 min prior to tracer application with 2-PMPA already under anesthesia.

Organs with the highest uptake were kidneys in baseline conditions (**[^18^F]­7**: 11.0 ± 2.4, **[^18^F]­8**: 13.2 ± 0.8) and the bladder including urine (**[^18^F]­7**: 12.8 ± 5.8, **[^18^F]­8**: 12.2 ± 4.4) for both tracers (Figure 6 and Supplementary Figure S15). In both organs, a rapid increase in radioactivity uptake with a constant SUV at 10–20 min after injection was observed due to clearance of radioactivity from other organs and endogenously expressed PSMA in the kidneys. Time-activity curves showed a fast washout from other nontarget organs such as the heart representing the blood pool, muscle, lung, and liver (Supplementary Figures S16 and S17). A fast increase in tumor uptake after tracer administration was observed, and the SUV reached a plateau after 10 min postinjection with a minor decrease over time. Tumor/blood and tumor/muscle ratios increased constantly over time (Figure 7). A variation in tumor uptake in individual animals was observed but could not be attributed to differences in tumor size. Maximum intensity projections of PET images (15–90 min) clearly visualized the tumor with both tracers and showed a significant decrease in tumor uptake when 2-PMPA was used as a blocking agent (Figure 6 and Supplementary Figures S16 and S17).

**Figure 6:**
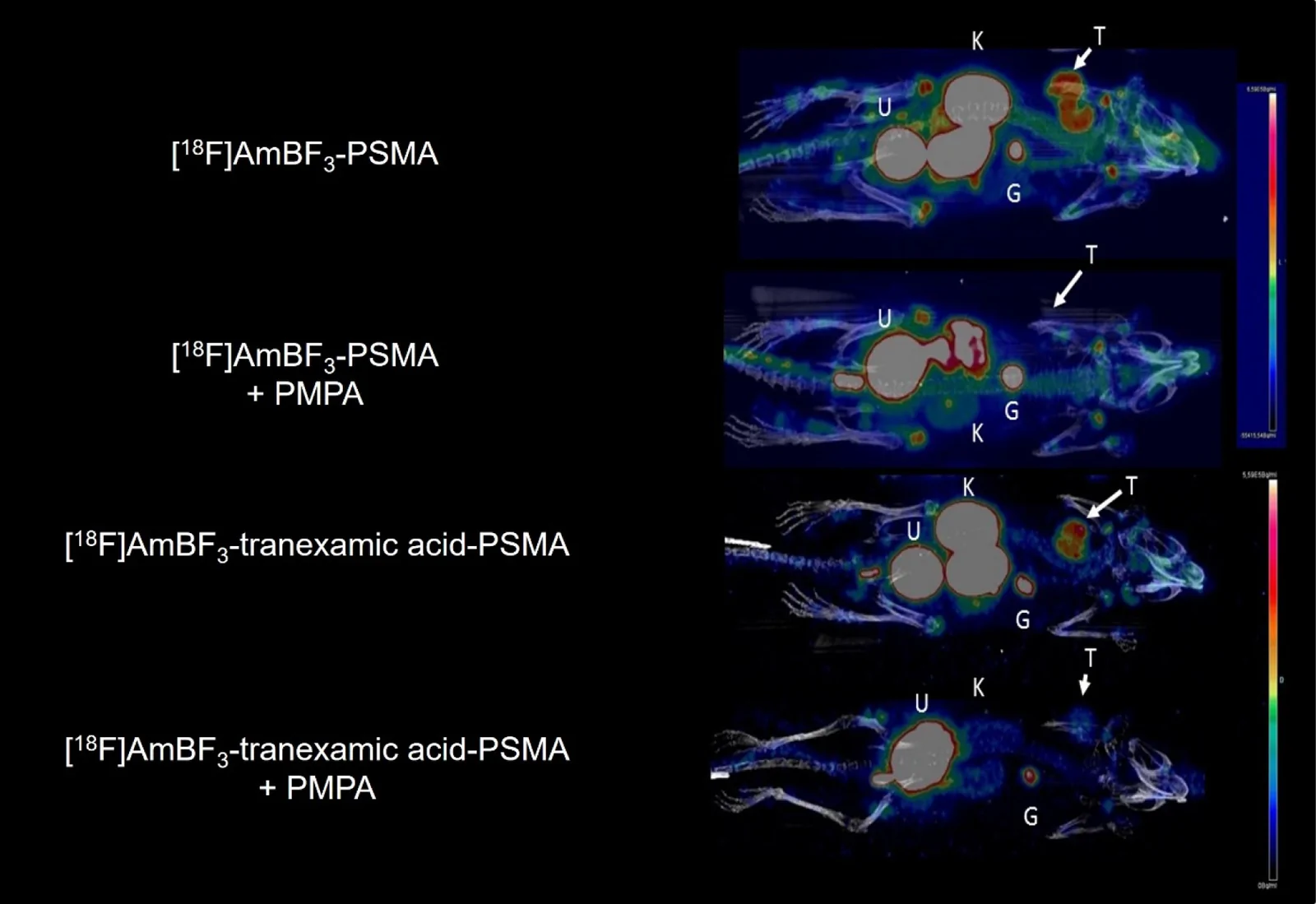
Maximum intensity projections of PET/CT at 15–90 min in C4-2 xenografts. Blocked with 2-PMPA 30 min prior to tracer injection. *T* = tumor, G = gallbladder, K = kidney, U = urine.

**Figure 7:**
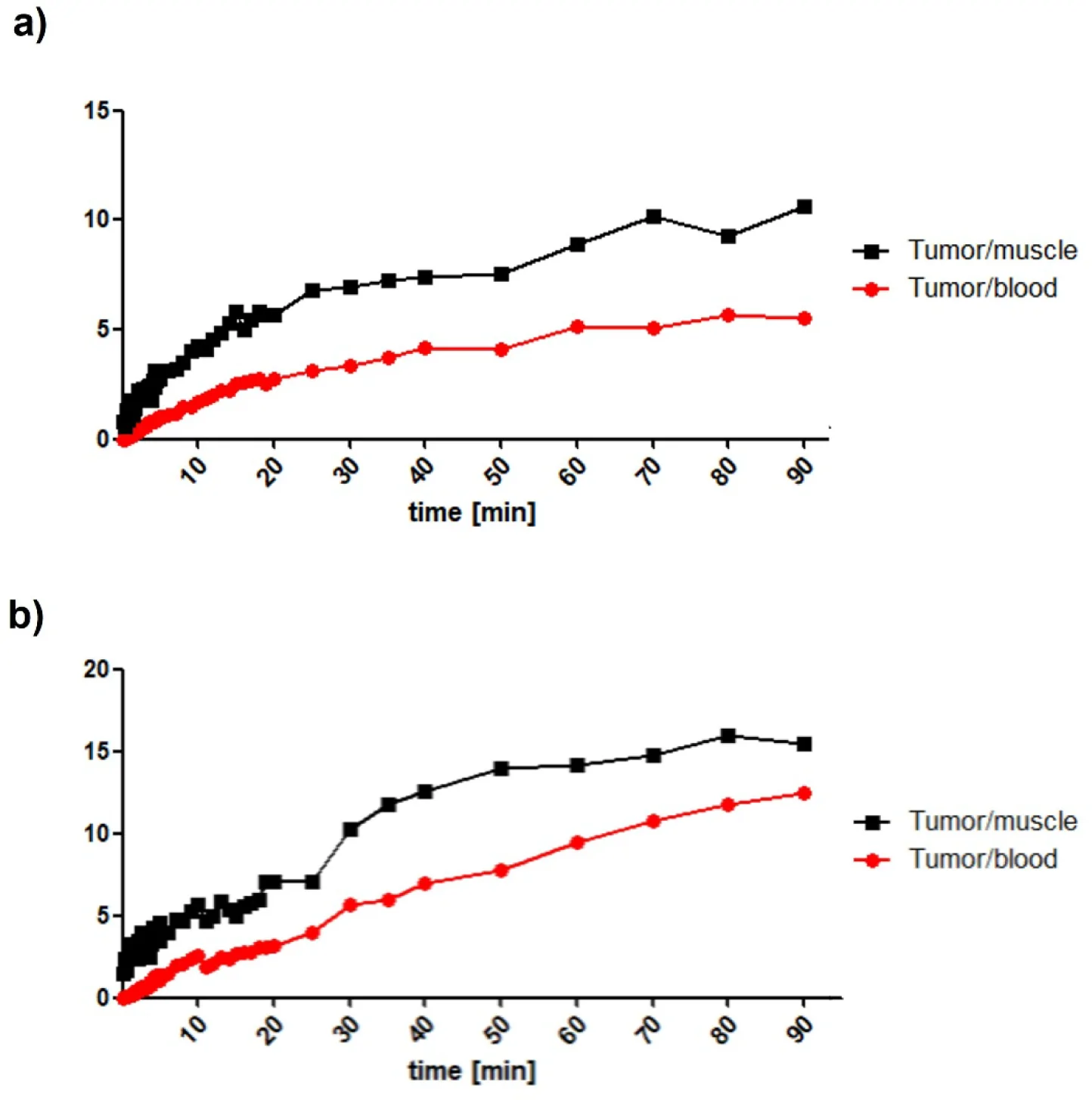
Tumor/muscle and tumor/blood ratios of a) [^18^F]­AmBF_3_-PSMA **[^18^F]­7** and b) [^18^F]­AmBF_3_-tranexamic acid-PSMA **[^18^F]­8**.

## 3 Discussion

The aim of the present study was to investigate the suitability of our previously developed prosthetic group [^18^F]­AmBF_3_-Tz (**[^18^F]­1**) for radiolabeling of low-molecular-weight peptidomimetics.^23^ Urea-based targeting of PSMA is a well-established procedure for visualization of prostate cancer both in preclinical and in clinical settings and therefore is an optimal candidate for the evaluation of our bioorthogonal labeling strategy. To modify the pharmacological properties, two different analogs of [^18^F]­AmBF_3_-PSMA were synthesized and evaluated *in vitro* and *in vivo*. To investigate the effect of the TCO-tetrazine bioorthogonal pair on PSMA binding, structure **7** was chosen. To modify the hydrophilic character of **7** and to enhance PSMA binding by the addition of a ring structure, a tranexamic acid linker was introduced resulting in compound **8**. Synthesis of the TCO-functionalized PSMA derivatives was achieved in a two- or five-step synthesis at decent yields (41–71%).

The postradiolabeling conjugation of **[^18^F]­1** to TCO-bearing precursors **2** and **6** for **[^18^F]­7** and **[^18^F]­8**, respectively, resulted in several isomeric products with similar chromatographic properties to the desired product and therefore could not be purified by solid-phase extraction.^23^ This effect has also been reported for gallium-68-labeled peptides^32^ and was recently discussed by our group.^29^ To avoid extensive HPLC work-up, we carried out the IEDDA reaction before radiolabeling. The resulting preconjugated precursors enabled SPE purification, but the overall radiochemical yields remained modest (5–33%) and substantially below the 33 ± 5% yield of **[^18^F]­1** for the final tracers. Thus, the present results establish chemical feasibility rather than clinical-scale production readiness. For translation, optimization would need to address precursor concentration, reagent delivery, heat and pH control, and recovery losses. Automated microfluidic or cassette-based systems could improve mixing and temperature control in the 15–30 μL reaction volume, reduce operator dependence, and support more reproducible production. The two-step radiolabeling approach produced **[^18^F]­7** and **[^18^F]­8** with lower molar activities (A_m_ between 0.2 and 0.3 GBq/μmol) than the direct [^18^F]­fluorination of the preconjugated precursors **7** and **8** (A_m_ between 9.2 and 16.3 GBq/μmol). In general, lower molar activity may increase the amount of nonradioactive tracer administered, potentially affecting receptor occupancy and quantitative imaging interpretation favoring the direct [^18^F]­fluorination approach in our case. Additional optimization of precursor design and cartridge purification would also be required to further improve decay-corrected yield and molar activity.

Cellular uptake and binding specificity were determined in a PSMA-expressing human prostate carcinoma C4-2 cell model in vitro; uptake was challenged by coincubation with 2-PMPA, confirming specific uptake of the tracers in C4-2 cells. No independent competitive binding assay or K_i_/IC_50_ determination was performed in the present study.

Nonreceptor-mediated uptake of radioactivity was also observed in the gallbladder and intestines for both tracers, as the uptake was not decreased by administration of the blocking agent. Similar hepatobiliary activity has been observed previously with aromatic AmBF_3_-PSMA compounds.^28^ However, no radio-HPLC analysis of plasma, bile, or excreted metabolites was performed in the present study. Therefore, the proposed contribution of hydrophobic tetrazine-TCO reaction products or lipophilic radiometabolites remains speculative. Metabolite analysis is required to test this interpretation.

The average tumor uptake values were 7.5 ± 0.7%ID/g for **[^18^F]­7** and 12.9 ± 4.8%ID/g for **[^18^F]­8**, compared with 8.0%ID/g reported for [^18^F]­PSMA-1007 after 60 min.^20^ No statistically significant difference in absolute tumor uptake was observed between **[^18^F]­7** and **[^18^F]­8** (n = 6, p = 0.2), although the mean value was numerically higher for **[^18^F]­8**. Tumor/blood (5.2 for **[^18^F]­7** and 9.5 for **[^18^F]­8**) and tumor/muscle (8.9 for **[^18^F]­7** and 14.2 for **[^18^F]­8**) ratios after 60 min were more favorable for **[^18^F]­8** in this data set, but these ratio-based findings should be interpreted in view of the small group sizes. Accordingly, the more favorable performance of [^**18**^ **F]­8** refers specifically to the tumor-to-blood and tumor-to-muscle ratios, not to a statistically significant increase in absolute tumor uptake.

The reported tumor uptake values for [^18^F]­DCFPyL and other [^18^F]­trifluoroborate-PSMA-targeting tracers in an LNCaP tumor model (4.4–16.7%ID/g at 60 min) are comparable to **[^18^F]­7** and **[^18^F]­8**.^28^ C4-2 cells are a derivative of LNCaP, and therefore, a direct comparison of uptake is possible.^33^ In particular, the clinically relevant [^18^F]­DCFPyL showed a tumor uptake of 11.6%ID/g, indicating that **[^18^F]­7** and **[^18^F]­8** achieved tumor accumulation within a similar performance range. Therefore, these findings support the potential of [^**18**^ **F**]­7 and [^**18**^ **F**]­8 as PSMA-targeted imaging agents, while further optimization may enhance their pharmacokinetics and imaging properties.

## 4 Conclusion

Conjugation of AmBF_3_-Tz to TCO-functionalized PSMA derivatives prior to radiolabeling simplified purification and enabled the preparation of fluorine-18-labeled tracers with reproducible radiochemical purity greater than 95% following SPE purification and with higher molar activity. Both **[^18^F]­7** and **[^18^F]­8** visualized PSMA-expressing C4-2 xenografts. **[^18^F]­8** showed more favorable tumor-to-background ratios, whereas its absolute tumor uptake was not significantly different from that of **[^18^F]­7**. This proof-of-concept study supports AmBF_3_-Tz as a strategy for developing fluorine-18-labeled low-molecular-weight PET probes and warrants further optimization, including improved radiochemical yield, molar activity, microscale reproducibility, and metabolite characterization, before clinical translation.

## 5 Methods and Materials

Commercially available chemicals were used without further purification unless otherwise stated. 2,5-Dioxopyrrolidin-1-yl 2-(4-(1,2,4,5-tetrazin-3-yl)­phenyl)­acetate was purchased from Jena Bioscience (Jena, Germany). 2-(Iodomethyl)-4,4,5,5-tetramethyl-1,3,2-dioxaborolane was purchased from Enamine (Kyiv, Ukraine). ((5-Amino-1-carboxypentyl)­carbamoyl)­glutamic acid was purchased from ABX Advanced Biochemical Compounds (Radeberg, Germany). Di-*tert*-butyl­((6-amino-1-(*tert*-butoxy)-1-oxohexan-2-yl)­carbamoyl)­glutamate was purchased from FutureChem (Seoul, South Korea), and 4-(((((9H-fluoren-9-yl)­methoxy)­carbonyl)­amino)­methyl)­cyclohexane-1-carboxylic acid was purchased from TCI Chemicals (Eschborn, Germany). (*E*)-Cyclooct-4-en-1-yl (2,5-dioxocyclopentyl) carbonate was purchased from Click Chemistry Tools (Scottsdale, AZ, USA). Anhydrous acetonitrile (99.8%), *N*,*N*-dimethylethylenediamine (95%), *N*,*N*-dimethylformamide (≥99%), and *N*,*N*-diisopropylethylamine (95%) were purchased from Sigma-Aldrich (St. Louis, MO, USA).

Alternative route: *trans*-4-((*tert*-Butoxycarbonyl)­amino)­cyclohexane-1-carboxylic acid and *N*,*N*,*N′*,*N′*-tetramethyl-*O*-(*N*-succinimidyl)­uronium tetrafluoroborate were bought from TCI Europe N.V. (Haven, Belgium), and TCO-*N*-hydroxysuccinimide (NHS) ester (CAS: 1191901-33-3) was bought from BroadPharm (San Diego, USA). The nuclear magnetic resonance (NMR) spectra were either recorded with a Bruker Ultrashield TH 400 plus at 400 MHz, or with a Bruker Avance-III spectrometer operating at 500.06 MHz (^1^H), 125.75 MHz (^13^C) equipped with Smartprobe: BB/1H was used for acquiring ^1^H, ^13^C, COSY, multiplicity edited HSQC (CH and CH_3_ positive, CH_2_ negative, both coupled and decoupled), HMBC and 1D-TOCSY. The measured NMR spectra were calibrated using the residual solvent signal as an internal standard. High-resolution mass spectroscopy (HRMS) was carried out with a UPLC-ACQUITY Waters RDa detector (electrospray ionization (ESI) with a time-of-flight (TOF) detector).

Sep-Pak C18-Light cartridges were purchased from Waters (Milford, MA, USA) and PS-HCO_3_-cartridges from Macherey-Nagel (Düren, Germany). No-carrier-added [^18^F]­fluoride was produced at the Department of Chemistry, University of Helsinki, with an IBA Cyclone 10/5 medical cyclotron from oxygen-18 in an ^18^O­(*p*,*n*)^18^F reaction. Oxygen-18-enriched water (98%) was purchased from Rotem Industries Limited (Arava, Israel).

HPLC analysis was performed on a Shimadzu UFLC HPLC system equipped with a DGU-20A5 degasser, an SPD-M20A UV detector, an LC-20AT pump system, a CBM-20A communication BUS module, a CTO-20AC column oven, and a Scionix Holland scintillation detector with a 51 BP 51/2 NaI­(Tl) crystal.

Method 1: Phenomenex Kinetex column (C18, 5 μm, 10 × 250 mm; Torrance, United States) (flow rate: 2.5 mL/min; solvent A: 0.1% trifluoroacetic acid (TFA) in Milli-Q water (mQ); solvent B: 0.1% TFA in acetonitrile; elution gradient: 95% to 5% A in 15 min; temperature: 50 °C).

Method 2: Phenomenex Kinetex column (C18, 5 μm, 10 × 250 mm) (flow rate: 2.5 mL/min; solvent A: 0.1% TFA in mQ; solvent B: 0.1% TFA in acetonitrile; elution gradient: 80% to 50% A in 20 min, 50% to 80% A in 20 to 25 min, and 80% A in 25 to 30 min; temperature: 50 °C).

PSMA analogues were analyzed by HPLC Method 1. AmBF_3_-Tz (**1**) and IEDDA conjugates were analyzed by HPLC Method 2.

Liquid chromatography-mass spectrometry (LC-MS) was performed on an Agilent Technologies 1200 series HPLC-DAD system with an Agilent Technologies 6120 Quadrupole LC/MS detector in scan mode (scan range 50–2000 *m*/*z*) and a Waters Atlantis T3 column (3 μm, 4.6 × 150 mm; Eschborn, Germany) (solvent A: 0.1% formic acid (FA) in mQ; solvent B: 0.1% FA in acetonitrile; elution gradient 90% to 10% A in 20 min), fragmentor voltage of 90 V, positive-ionization mode, column temperature of 50 °C, gas temperature of 300 °C, gas flow of 11 L/min, and nebulizer pressure of 15 psi.

NMR spectra were acquired with a Bruker UltraShield TH 400 Plus instrument at 400 MHz.

Thin-layer chromatography (TLC) and radio-TLC were performed with a mobile phase of acetonitrile/mQ 80:20 (v:v) on a normal-phase silica gel plate and were analyzed with a Fujifilm FLA 5100 scanner (Fujifilm Life Sciences, Cambridge, MA, USA).

An automatic γ-counter (1480 Wizard 3, PerkinElmer, Waltham, United States) was used to quantify radioactivity in *in vitro/vivo* samples.

Clinical [^18^F]­PSMA-1007 batch, obtained from the HUS Diagnostic Center, Cyclotron Unit (Helsinki, Finland), was manufactured by good manufacturing practices (GMP) according to draft of the Eur. Ph. Monograph: 07/2021:3116PSMA-1007 (^18^F) injection.

### 5.1 Synthesis of AmBF_3_-Tz ((((2-(2-(4-(1,2,4,5-Tetrazin-3-yl)­phenyl)­acetamido)­ethyl)­dimethylammonio)­methyl)­trifluoroborate) (**1**)

The synthesis of AmBF_3_-Tz (**1**) has been reported recently by our group.^23^ In brief, **1** was synthesized in a four-step synthesis starting from the commercially available tetrazine NHS ester (2,5-dioxopyrrolidin-1-yl 2-(4-(1,2,4,5-tetrazin-3-yl)­phenyl)­acetate), which was conjugated with *N* ^1^,*N* ^1^-dimethylethane-1,2-diamine. Following conjugation with 2-(iodomethyl)-4,4,5,5-tetramethyl-1,3,2-dioxaborolane in dry acetonitrile and subsequent fluorination of the pinacol ester under acidic conditions, the final product (((2-(2-(4-(1,2,4,5-tetrazin-3-yl)­phenyl)­acetamido)­ethyl)­dimethylammonio)­methyl)­trifluoroborate was synthesized in an overall yield of 39.4% (n = 6).

^1^H NMR (400 MHz, CD_3_CN) δ 10.30 (s, 1H), 8.54 (d, *J* = 8.5 Hz, 2H), 7.58 (d, *J* = 8.6 Hz, 2H), 3.68–3.56 (m, 4H), 3.34 (t, *J* = 6.7 Hz, 2H), 3.01 (s, 6H), 2.38 (s, 2H). ^11^B NMR (128 MHz, CD_3_CN) δ 103.1, 102.8, 102.4, 102.0. ^19^F NMR (376 MHz, CD_3_CN) δ −138.8, −138.9, −139.0, −139.2. ^13^C NMR (101 MHz, CD_3_CN) δ 171.5, 167.3, 159.0, 142.0, 131.8, 131.4, 129.1, 118.3, 65.4, 54.3, 43.4, 34.8, 1.3. HRMS calculated for C_15_H_21_BF_3_N_6_O^+^ [M + H]^+^ 369.18165 *m*/*z*, found C_15_H_21_BF_3_N_6_O^+^ [M+H^+^] 369.18134 *m*/*z* (mass error −0.85 ppm).

### 5.2 Synthesis of PSMA-TCO ((*E*)-((1-Carboxy-5-(((cyclooct-4-en-1-yloxy)­carbonyl)­amino)­pentyl)­carbamoyl)­glutamic acid) (**2**)

To TCO-NHS ((*E*)-cyclooct-4-en-1-yl (2,5-dioxocyclopentyl) carbonate) (4.5 mg, 17.0 μmol) in dry *N*,*N*-dimethylformamide (DMF, 300 μL) and *N*,*N*-diisopropylethylamine (DIPEA, 3.2 mg, 25.0 μmol) under an argon atmosphere, PSMA-amine (((5-amino-1-carboxypentyl)­carbamoyl)­glutamic acid) (5 mg, 15.7 μmol) in dry DMF (250 μL) was added dropwise and stirred overnight. The yielded PSMA-TCO **2** was purified by HPLC to give 5.3 mg (71%) with >95% purity (HPLC). LC-MS (+) *m*/*z* (%) = 472.5 (75) [M + H]^+^, 320.3 (100) [M – TCO-formate]^+^ t_*R*_ = 10.3 min. ^1^H NMR (400 MHz, methanol-d_4_) δ (ppm) = 5.59 (m, 1H), 5.50 (m, 1H), 4.31 (m, 1H), 4.25 (s, 1H), 3.31 (s, 2H), 3.06 (m, 2H), 2.41 (m, 2H), 2.33 (m, 2H), 2.33 (m, 2H), 2.15 (m, 2H), 1.94 (m, 6H), 1.68 (m, 4H), 1.40 (m, 4H), 1.29 (m, 2H). ^13^C NMR (101 MHz, methanol-d_4_) δ (ppm) = 136.1, 133.8, 81.6, 54.1, 53.6, 42.2, 39.7, 35.2, 33.5, 33.3, 32.1, 31.2, 30.6, 29.0, 23.9.

### 5.3 Synthesis of PSMA-Tranexamic Acid-TCO ((*E*)-((1-Carboxy-5-(4-((((cyclooct-4-en-1-yloxy)­carbonyl)­amino)­methyl)­cyclohexane-1-carboxamido)­pentyl)­carbamoyl)­glutamic acid) (**6**)

Di-tert-butyl­((6-(4-(((((9H-fluoren-9-yl)­methoxy)­carbonyl)­amino)­methyl)­cyclohexane-1-carboxamido)-1-(tert-butoxy)-1-oxohexan-2-yl)­carbamoyl)­glutamate (**3**)

To (2-(1H-benzotriazol-1-yl)-1,1,3,3-tetramethyluronium hexafluorophosphate (HBTU, 76.6 mg, 202.0 μmol) and DIPEA (26.4 mg, 206.0 μmol) in dry DMF (400 μL), fluorenylmethoxycarbonyl (Fmoc)-protected Fmoc-tranexamic acid (4-(((((9H-fluoren-9-yl)­methoxy)­carbonyl)­amino)­methyl)­cyclohexane-1-carboxylic acid) (78 mg, 206.0 μmol) was added in dry DMF (600 μL) and stirred for 10 min under an argon atmosphere. Di-*tert*-butyl ((6-amino-1-(*tert*-butoxy)-1-oxohexan-2-yl)­carbamoyl)­glutamate (25 mg, 52.0 μmol) was added in dry DMF (400 μL) and stirred under argon for 2 h. LC-MS (+) *m*/*z* (%) = 850.1 (4) [M + H]^+^, 872.1 (3) [M + Na]^+^ t_*R*_ = 18.5 min.

Di-tert-butyl­((6-(4-(aminomethyl)­cyclohexane-1-carboxamido)-1-(tert-butoxy)-1-oxohexan-2-yl)­carbamoyl)­glutamate (**4**)

Without further purification, 1.4 mL of piperidine was added to **3** and stirred for >10 min at room temperature (RT). The solvents were evaporated, and the product was extracted with 5 mL of ethyl acetate and washed with 3 × 2 mL of brine solution. LC-MS (+) *m*/*z* (%) = 628.0 (100) [M + H]^+^ t_*R*_ = 11.3 min.

((5-(4-(Aminomethyl)­cyclohexane-1-carboxamido)-1-carboxypentyl)­carbamoyl)­glutamic acid (**5**)

Without further purification, **4** was dissolved in 3 mL of dichloromethane/TFA (1:1) and stirred for 90 min at RT. The product was purified by HPLC (t_*R*_ = 7.2 min), yielding 10.2 mg (43%). LC-MS (+) *m*/*z* (%) = 459 (100) [M + H]^+^, t_*R*_ = 2.6 min.

(E)-((1-Carboxy-5-(4-((((cyclooct-4-en-1-yloxy)­carbonyl)­amino)­methyl)­cyclohexane-1-carboxamido)­pentyl)­carbamoyl)­glutamic acid (**6**)

To TCO-NHS (13 mg, 49.0 μmol) in dry DMF (600 μL) and DIPEA (8.9 mg, 70.0 μmol), **5** was added dropwise without further purification in 250 μL of dry DMF and stirred overnight under an argon atmosphere.

Product **6** was purified by HPLC (t_*R*_ = 4.5 min) yielding 5.63 mg (41%). LC-MS (+) *m*/*z* (%) = 611 (100) [M + H]^+^ t_*R*_ = 11.4 min. ^1^H NMR (400 MHz, methanol-d_4_) δ (ppm) = 5.61 (m, 1H), 5.52 (m, 1H), 4.32 (m, 2H), 4.26 (m, 1H), 3.17 (m, 2H), 3.01 (s, 1H), 2.92 (m, 2H), 2.88 (s, 1H), 2.43 (m, 2H), 2.34 (m, 2H), 2.15 (m, 2H), 1.98 (m, 4H), 1.80 (m, 5H), 1.70 (m, 4H), 1.51 (m, 2H), 1.44 (m, 5H), 0.98 (m, 2H), ^13^C NMR (101 MHz, methanol-d_4_) δ (ppm) = 136.1, 133.8, 53.9, 53.5, 46.5, 42.2, 39.9, 39.7, 39.1, 35.2, 33.5, 33.2, 32.1, 31.0, 30.2, 30.0, 28.9, 26.5, 23.9.

For *in vivo* work, **6** was synthesized as described above. Further optimization resulted in a different reaction pathway, as described here:

### 5.4 Synthesis of PSMA-Tranexamic Acid-TCO by an Alternative Route

Di-tert-butyl-(((S)-1-(tert-butoxy)-6-((1r,4S)-4-(((tert-butoxycarbonyl)­amino)­methyl)­cyclohexane-1-carboxamido)-1-oxohexan-2-yl)­carbamoyl)-l-glutamate. *trans*-4-((*tert*-Butoxycarbonyl)­amino)­cyclohexane-1-carboxylic acid (95 mg, 0.37 mmol) and *N*,*N*,*N′*,*N′*-tetramethyl-*O*-(*N*-succinimidyl)­uronium tetrafluoroborate (120 mg, 0.4 mmol) were dissolved to dry *N,N*-dimethylformamide (0.7 mL) under a nitrogen balloon. Diisopropylethylamine (70 μL, 0.40 mmol) was added and the mixture was stirred at RT for 2.5 h. (*S*)-Di-*tert*-butyl-2-(3-((*S*)-6-amino-1-*tert*-butoxy-1-oxohexan-2-yl)­ureido)­pentanedioate (150 mg, 0.3 mmol) and DIPEA (70 μL, 0.40 mmol) were dissolved in dry *N,N*-dimethylformamide (0.7 mL) and added to the previous mixture. After continuous stirring overnight, ethyl acetate (20 mL) was added, and the solution was extracted twice with 1 M NaOH (10 mL each) and subsequently twice with 1 M HCl (10 mL each). The organic phase was dried over sodium sulfate and evaporated. The thick oil could be used as such for the next steps, but the oil was dissolved in dichloromethane (0.5 mL) and diluted with diethyl ether (5 mL) and placed at −18 °C overnight. The liquid was decanted and the white powder was washed with cold diethyl ether (5 mL). Yield: 87 mg (39%) containing minor impurities, mp = 90–93 °C. ^1^H NMR (500.13 MHz, CDCl_3_) δ 6.05 (s, 1H), 5.40 (d, J = 6.6 Hz, 1H), 5.39 (d, J = 6.1 Hz, 1H), 4.62 (br, 1H), 4.35–4.29 (m, 1H), 4.28–4.22 (m, 1H), 3.27–3.14 (m, 2H), 3.02–2.91 (m, 2H), 2.38–2.23 (m, 2H), 2.12–2.00 (m, 2H), 1.91–1.79 (m, 5H), 1.78–1.70 (m, 1H), 1.64–1.53 (m, 1H), 1.45 (m, 41H), 1.38–1.28 (m, 2H), 0.96 (m, 2H). ^13^C NMR (125.79 MHz, CDCl_3_) δ 176.3, 172.7, 172.6, 172.5, 157.3, 156.1, 82.2, 81.8, 80.7, 79.2, 53.5, 53.1, 46.8, 45.5, 38.9, 37.8, 32.6, 31.7, 30.03, 29.99, 29.2, 29.1, 28.5, 28.4, 28.2, 28.1, 22.6. HR-ESI-MS (m/z) [M + H]+ calcd. for C_37_H_67_N_4_O_10_^+^ 727.4852 found 727.4880.

*(E)-*((1-Carboxy-5-(4-((((cyclooct-4-en-1-yloxy)­carbonyl)­amino)­methyl)­cyclohexane-1-carboxamido)­pentyl)­carbamoyl)­glutamic acid (**6**). Di-tert-butyl-(((*S*)-1-(tert-butoxy)-6-((1*r*,4*S*)-4-(((tert-butoxycarbonyl)­amino)­methyl)­cyclohexane-1-carboxamido)-1-oxohexan-2-yl)­carbamoyl)-l-glutamate (20.6 mg, 0.028 mmol) was dissolved in dichloromethane (1.0 mL) followed by addition of trifluoroacetic acid (0.4 mL). The mixture was kept under nitrogen and stirred overnight at RT. The solution was evaporated and re-evaporated thrice from acetonitrile (2 mL each). *trans*-Cyclooctene NHS ester (5.7 mg, 0.036 mmol), diisopropylethylamine (24 μL, 0.058 mmol), and dry *N,N*-dimethylformamide (1.0 mL) were added, and the mixture was protected from light and stirred under nitrogen at RT overnight. The solution was diluted with water (20 mL) and pushed through two in-series strong cation exchange cartridges (Chromafix PS-H^+^ light) and washed with water (10 mL). The combined aqueous solution was divided into three Aminopropyl Plus cartridges, which were preconditioned with acetonitrile (6 mL) followed by water (20 mL). The cartridge stacks were flushed with water (10 mL each) and acetonitrile (6 mL each) and then eluted with 1% formic acid in methanol (6 mL). The organics were combined, and chloroform was added to the solution (20 mL). The chloroform was evaporated, and the treatment with chloroform was repeated twice before evaporating to dryness yielding a colorless oil. Coevaporation with chloroform is important as concentration with formic acid leftovers may lead to degradation of the product. Yield: 7.3 mg (43%). ^1^H NMR (500.13 MHz, MeOD) δ 5.65–5.54 (m, 1H), 5.51–5.43 (m, 1H), 4.34–4.23 (m, 3H), 3.18–3.12 (m, 2H), 2.92 (bd, 2H), 2.44–2.38 (m, 2H), 2.37–2.29 (m, 2H), 2.14–2.07 (m, 2H), 2.03–1.77 (m, 10H), 1.77–1.27 (m, 12H), 1.0–0.90 (m, 2H). ^13^C NMR (125.79 MHz, MeOD) δ 179.1, 176.4, 175.9, 175.80, 160.1, 158.9, 136.0, 133.6, 81.6, 54.0, 53.5, 47.8, 46.5, 42.2, 39.9, 39.7, 39.1, 35.2, 33.5, 33.2, 32.1, 31.1, 31.0, 30.2, 30.0, 28.9, 23.9. HR-ESI-MS [M-H]^−^ anal. calcd. for C_29_H_45_N_4_O_10_^–^ 609.3141, found: 609.3142.

### 5.5 IEDDA Conjugation of AmBF_3_-Tz (**1**) with PSMA-*trans*-Cyclooctene (**2**) or PSMA-Tranexamic Acid-*trans*-Cyclooctene (**6**)

**2** or **6** was added to **1** in 20 μL of pyridazine-HCl buffer (pH 2) in equimolar amounts in mQ (800 μL). The reaction was heated to 60 °C for 20 min. The product was purified by HPLC and afforded **7** (49%) (t_*R*_ = 7.9 min) with a purity of >95% (HPLC). LC-MS (+) *m*/*z* (%) = 811 (100) [M + H]^+^ t_*R*_ = 8.8 min (NMR Supplementary Figure S6) or **8** (48%) (t_*R*_ = 9.5 min); LC-MS (+) *m*/*z* (%) = 950 (100) [M + H]^+^ t_*R*_ = 9.5 min (NMR Supplementary Figure S7).

### 5.6 Radiochemistry

#### 5.6.1 Synthesis of [^18^F]­AmBF_3_-Tz (**[^18^F]­1**)

[^18^F]­Fluoride (1–10 GBq) was eluted from a PS-HCO_3_ cartridge with 100 μL of 0.9% NaCl solution and evaporated to a volume of 10–15 μL at 100 °C. **1** (100 nmol) in 10 μL of pyridazine HCl buffer (pH = 2) was added, and the resulting solution was heated to 85 °C for 10 min. The crude reaction was analyzed by radio-HPLC and yielded **[^18^F]­1** with a nonisolated RCY of 33 ± 5%, n = 6.

### 5.7 Synthesis of [^18^F]­AmBF_3_-PSMA (**[^18^F]­7**) and [^18^F]­AmBF_3_-Tranexamic Acid-PSMA (**[^18^F]­8**) via Radiolabeling of **1** Prior to the IEDDA Conjugation

Without further purification of [^**18**^ **F]­1** in residual pyridazine HCl buffer volume (0.8–8 GBq), **2** or **6** (100 nmol) was added in 200 μL of mQ, and the reaction was heated to 60 °C for 20 min. After dilution with 10 mL of mQ, the activity was loaded on a preconditioned (1 mL ethanol +10 mL mQ) C18 cartridge and washed with an additional 40 mL of mQ before elution with 800 μL of 50% EtOH in 1 × phosphate-buffered saline (PBS). HPLC purification afforded **[^18^F]­7** (decay-corrected RCY: 0.7 ± 0.4%, n = 3) with a molar activity of 0.2 ± 0.02 GBq/μmol at the end of synthesis (EOS) and **[^18^F]­8** (decay-corrected RCY: 9.8 ± 0.4%, n = 3) with a molar activity of 0.3 ± 0.09 GBq/μmol at EOS.

### 5.8 Synthesis of [^18^F]­AmBF_3_-PSMA (**[^18^F]­7**) and [^18^F]­AmBF_3_-Tranexamic Acid-PSMA (**[^18^F]­8**) via Direct ^18^F-Fluorination

[^18^F]­Fluoride (13–21 GBq) was eluted from a PS-HCO_3_ cartridge with 100 μL of 0.9% NaCl solution and evaporated to a volume of 10–15 μL at 100 °C. Precursor **7** or **8** (100 nmol) in 10 μL of pyridazine HCl buffer (pH 2) was added, and the resulting solution was heated to 85 °C for 10 min. After dilution with 10 mL of mQ, fluorine-18 was loaded on a preconditioned C18 cartridge and washed with an additional 40 mL of mQ before elution with 400 μL of 50% ethanol/1 × PBS affording [^**18**^ **F]­7** (decay-corrected RCY: 5.2 ± 1%, n = 3) with a molar activity of 9.2 ± 3.8 GBq/μmol at EOS and [^**18**^ **F]­8** (decay-corrected RCY: 11.8 ± 3.1%, n = 3) with a molar activity of 16.3 ± 4.3 GBq/μmol at EOS.

### 5.9 Lipophilicity

1-Octanol (2.0 mL) and 0.02 M PBS (2.0 mL) were pipetted into a 15 mL polypropylene centrifuge tube containing [^**18**^ **F]­7** or [^**18**^ **F]­8** in PBS. The tubes were mechanically shaken for 10 min and centrifuged (5 min, 1000 × *g*). 0.5 mL of each phase, aqueous and organic, was transferred into preweighed sample tubes, and the amount of radioactivity was determined by a 1480 Wallac Wizard 3” (PerkinElmerTM Life Sciences, Waltham, MA, USA) gamma counter for 60 s per sample (n = 3). The distribution coefficient (log*D (pH 7.4)*) was calculated by using eq 1:

1 $$\log\left(D\right)=\log\left(\frac{A_{octanol}}{A_{buffer}}\right)$$

where *D* = the distribution coefficient, *A_octanol_* = radioactivity in the octanol phase, and *A_buffer_* = radioactivity in the buffer phase, with the logarithm taken to obtain log *D*.

### 5.10 Shelf-Life Stability

To determine the shelf stability of [^**18**^ **F]­7** or [^**18**^ **F]­8** formulated in 1 × PBS, pH 7.4, a 5 μL sample was analyzed by radio-TLC after storing at RT for 0.5, 1, 2, 3, 4, 5, and 6 h (n = 3).

### 5.11 Plasma Stability

For determination of plasma stability, 400 μL of human plasma (Finnish Red Cross Blood Service, permission 33/2018) was incubated with 400 μL (250 nM) of the [^**18**^ **F]­7** or [^**18**^ **F]­8** formulation at 37 °C (n = 3) in a Protein LoBind microtube (Eppendorf). After 0.5, 1, 2, 3, and 4 h, samples of 100 μL were removed from the mixture. Proteins were precipitated by adding 50 μL of acetonitrile and separated from the supernatant by centrifugation at 17,949 × *g*. The supernatant was (further) analyzed by radio-TLC.

### 5.12 *In Vitro* Evaluation

#### 5.12.1 Cell Culturing

Human prostate carcinoma C4-2 cells (ATCC CRL-3314) were cultured in Dulbecco’s Modified Eagle’s Medium (Gibco) supplemented with 20% F-12 medium (Sigma-Aldrich), 10% fetal bovine serum (Gibco), and 1% T-medium (Gibco). Cells were cultured at 37 °C in a humidified incubator containing 5% CO_2_ and a relative humidity of 95%. Cells grown to 80–90% confluence were used in both *in vitro* and *in vivo* experiments.

### 5.13 *In Vitro* Cell Uptake

C4-2 cells were plated on a 6-well plate (sterile clear-bottom TC-treated, Corning) (6 × 10^5^ cells/well) 24 h prior to the cell experiment. The medium was changed to a CO_2_-independent medium (Gibco, Carlsbad, CA, USA) 30 min prior to the experiment. The cells in each well were incubated in ambient air with 1 mL of 250 nM [^**18**^ **F]­7** or [^**18**^ **F]­8** (9–16 GBq/μmol) in the CO_2_-independent medium. Specific cellular uptake was determined by blocking the cells with 2-(phosphonomethyl)­pentanedioic acid (2-PMPA) (final concentration 0.4 mM, Sigma). All experiments were conducted at 37 °C. The incubation was terminated after 30, 60, and 120 min by the removal of incubation media and washing twice with 1 mL of ice-cold 1 × PBS for determining the radioactivity in the free fraction. The cells were subsequently incubated twice with 1 mL of glycine-hydrochloric acid buffer (50 mM; pH 2.8) for 5 min each to collect the membrane-bound fraction. After an additional washing step with 1 mL of ice-cold 1 × PBS, the cells were lysed with 2 × 1 mL of sodium hydroxide (1 M) and washed with 1 mL of ice-cold 1 × PBS, and the liquids were collected to determine the internalized fraction. The radioactivity in each fraction was measured with an automatic γ-counter. Cell uptake was calculated as the percentage of membrane-bound and internalized radioactivity of the total added radioactivity. All experiments were carried out in triplicate.

An identical uptake experiment was done with the clinical tracer [^18^F]­PSMA-1007 (1 mL, 250 nM (12 GBq/μmol)) for comparison.

### 5.14 *In Vivo* Evaluation

All animal studies were approved by the National Project Authorisation Board (formerly the Animal Experiment Board) (license number 12132/04.10.07/2017) and were carried out in accordance with the EU Directive 2010/EU/63 on the protection of animals used for scientific purposes. Mice were group-housed in standard polysulfone cages with aspen bedding and with pelleted food (Harlan) and tap water available *ad libitum*. Environmental conditions of a 12:12 light/dark cycle, temperature of 22 ± 1 °C, and relative humidity of 55 ± 15% were maintained throughout the study.

### 5.15 Xenografts

C4-2 tumor cells were harvested from cell culturing flasks using TrypLE Express Enzyme A (Life Technologies Gibco, Carlsbad, CA, USA), centrifuged, and resuspended in 1:1 (v/v) of 1 × Hanks’ Balanced Salt Solution (HBSS) and Matrigel (Matrigel Basement Membrane Matrix, LDEV-free ref# 354234, Corning) to a final concentration of 33 × 10^6^ cells/mL. Male CB-17 SCID (severe combined immunodeficiency; CB17/Icr-*Prkdc* ^*scid*^/Rj) mice (weighing 19–27 g, aged 6–8 weeks, Janvier Laboratories, Le Genest-Saint-Isle, France) were anesthetized with isoflurane (2.5% in medical air:oxygen carrier, 3:2, 1 L/min), shaved at the neck, and subcutaneously inoculated with 5 × 10^6^ C4-2 cells in 150 μL between the scapulae. The tumors were allowed to develop until they reached 4–8 mm in the largest dimension.

### 5.16 *Ex Vivo* Biodistribution

Biodistribution of **[^18^F]­7** or **[^18^F]­8** was determined in C4-2 tumor-bearing SCID mice, and the specificity of tracer uptake was challenged by blocking with an excess amount of 2-PMPA *in vivo*. The number of animals was set at n = 3 per condition. For blocking conditions, mice were injected with 100 μL of a 0.4 mM 2-PMPA solution (40 nmol) via the lateral tail vein 30 min before the subsequent administration of **[^18^F]­7** or **[^18^F]­8**.

The radiotracer, **[^18^F]­7** or **[^18^F]­8**, was formulated at a concentration of 0.01 nM in sterile 1 × PBS and intravenously administered at 100 μL (1 nmol) via the lateral tail vein. At 1 h postinjection, animals were sacrificed by CO_2_ asphyxiation followed by cervical dislocation, and organs of interest were dissected, blotted dry, and weighed. Radioactivity was measured with an automatic γ-counter, and the results were presented as the percent of the injected radioactivity dose per gram of tissue (%ID/g) standardized to radioactivity samples of the injection volume (n = 3).

### 5.17 PET/CT Imaging

PET/CT imaging of **[^18^F]­7** or **[^18^F]­8** was conducted in C4-2 tumor-bearing SCID mice, and the specificity of uptake was challenged by blocking with 2-PMPA. Each experiment was conducted in triplicate. For blocking, the mice were injected with 0.4 mM 2-PMPA (100 μL; 40 nmol) via the tail vein 30 min before the injection of **[^18^F]­7** or **[^18^F]­8**.

The radiotracer, **[^18^F]­7** or **[^18^F]­8**, was administered as a 0.01 mM solution in 1 × PBS (100 μL; 1 nmol, 1–5 MBq) via tail vein cannulation under anesthesia. The PET/CT images were acquired with the Molecubes β-Cube (PET) and X-Cube (CT) systems (MOLECUBES NV, Ghent, Belgium). Dynamic imaging was performed for 90 min with the following time frames: 30 × 10 s, 15 × 60 s, 4 × 300 s, and 5 × 600 s. PET/CT images were analyzed with Carimas v2.10 software (Turku PET Center, Turku, Finland). Regions of interest (ROIs) were defined for the tumor, heart, kidney, urinary bladder, bone, muscle, lung, liver, and gallbladder and presented as standardized uptake values (SUVs).

### 5.18 Statistical Analysis

The data is presented as mean ± SD, unless indicated otherwise. Differences were tested with a two-tailed Mann–Whitney U Test for significance unless indicated otherwise. Differences at a probability level (*p*) of 0.05 were considered statistically significant. GraphPad Prism 5.01 (La Jolla, CA, USA) for Windows was used for statistical analyses.

## Supplementary Material



## Notes

Supplementary Figures S1–S17, including ^1^H and ^13^C NMR spectra, COSY, HSQC, HMBC, and 1D TOCSY spectra, LC-MS analyses, HPLC and TLC analyses of radiolabeled compounds, biodistribution data, and SUV values for [^18^F]­AmBF_3_-PSMA and [^18^F]­AmBF_3_-tranexamic acid-PSMA (PDF)
