# Supplementary material for: Design and Preclinical Evaluation of Two Prostate-Specific Membrane Antigen-Targeting Trifluoroborate Derivatives Suitable for One-Step Radiofluorination
Source: ACS Omega. 2026 Oct 20;11(43):67448–60. doi: 10.1021/acsomega.6c08595 (PMC13737878; doi:10.1021/acsomega.6c08595)
Supplement: Supplementary file 1 [file ao6c08595_si_001.pdf]

Design and preclinical evaluation of two prostate-specific membrane antigen targeting trifluoroborate derivatives suitable for one-step radiofluorination

Andreas Paulus [1], Sofia Otaru [1], Risto Savela [2], Surachet Imlimthan [1], Tatsiana Auchynnikava [2], Dominika Novotná [2], Anisa Biti [1], Mirkka Sarparanta [1], Heidi Liljenbäck [2,3], Tatu Martinmäki [1], Alexander Popkov [4,5], Kim A Bergström [6], Anne Roivainen [2,3], Anu J. Airaksinen [2,*]

[1] Department of Chemistry, Radiochemistry, University of Helsinki, FI-00014 Helsinki, Finland
[2] Turku PET Centre, University of Turku and Turku University Hospital, FI-20520 Turku, Finland
[3] Turku Center for Disease Modeling, University of Turku, FI-20520 Turku, Finland
[4] Institute of Organic Chemistry, Johannes Kepler University Linz, A-4040 Linz, Austria
[5] Samo University, CZ-37001 České Budějovice, Czech Republic
[6] HUS Diagnostic Center, Helsinki University Hospital and University of Helsinki, FI-00290 Helsinki, Finland

Corresponding Author *: Anu J. Airaksinen, anu.airaksinen@utu.fi

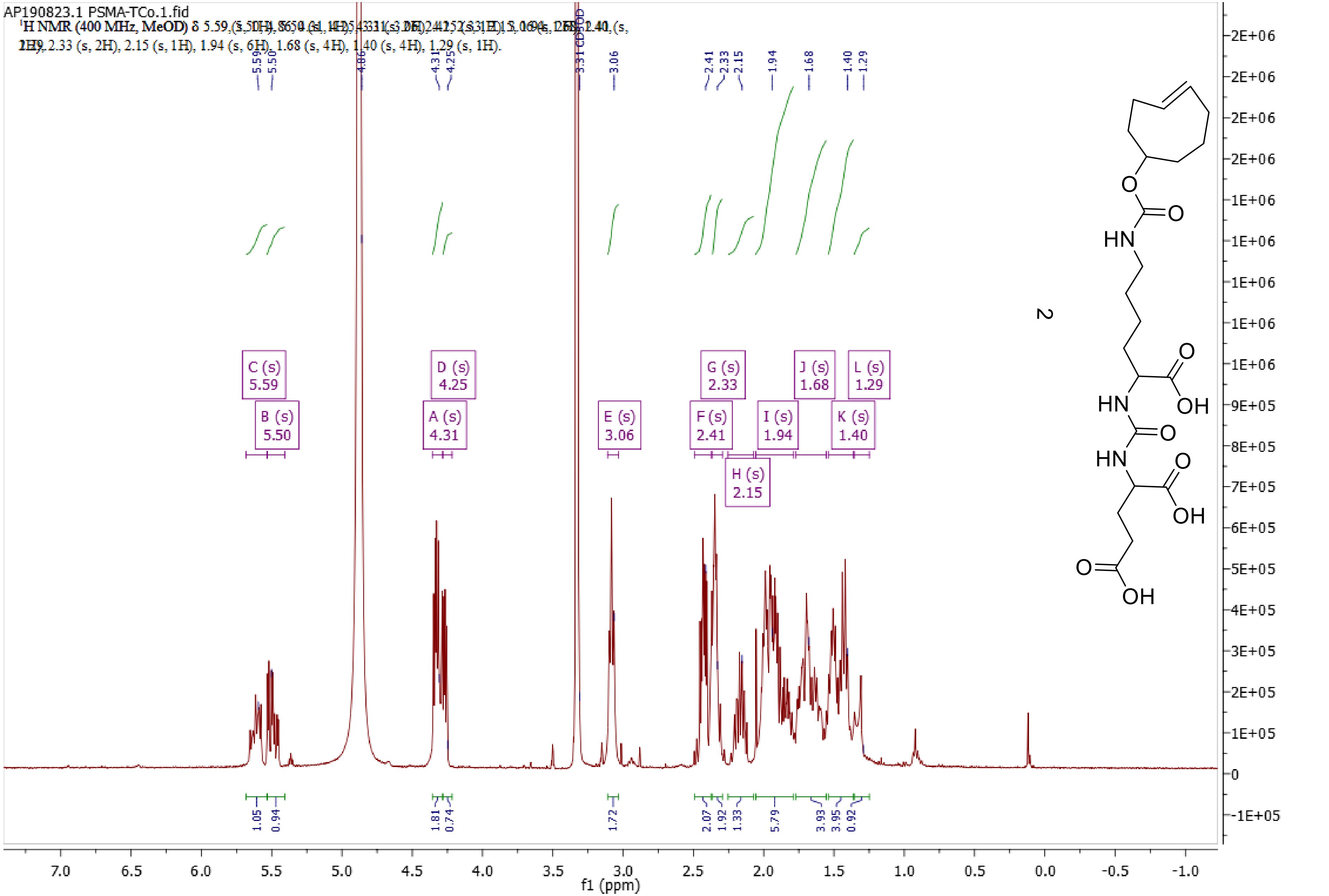

Supplementary Figure S1: H-NMR of PSMA-TCO (2) in d-Methanol

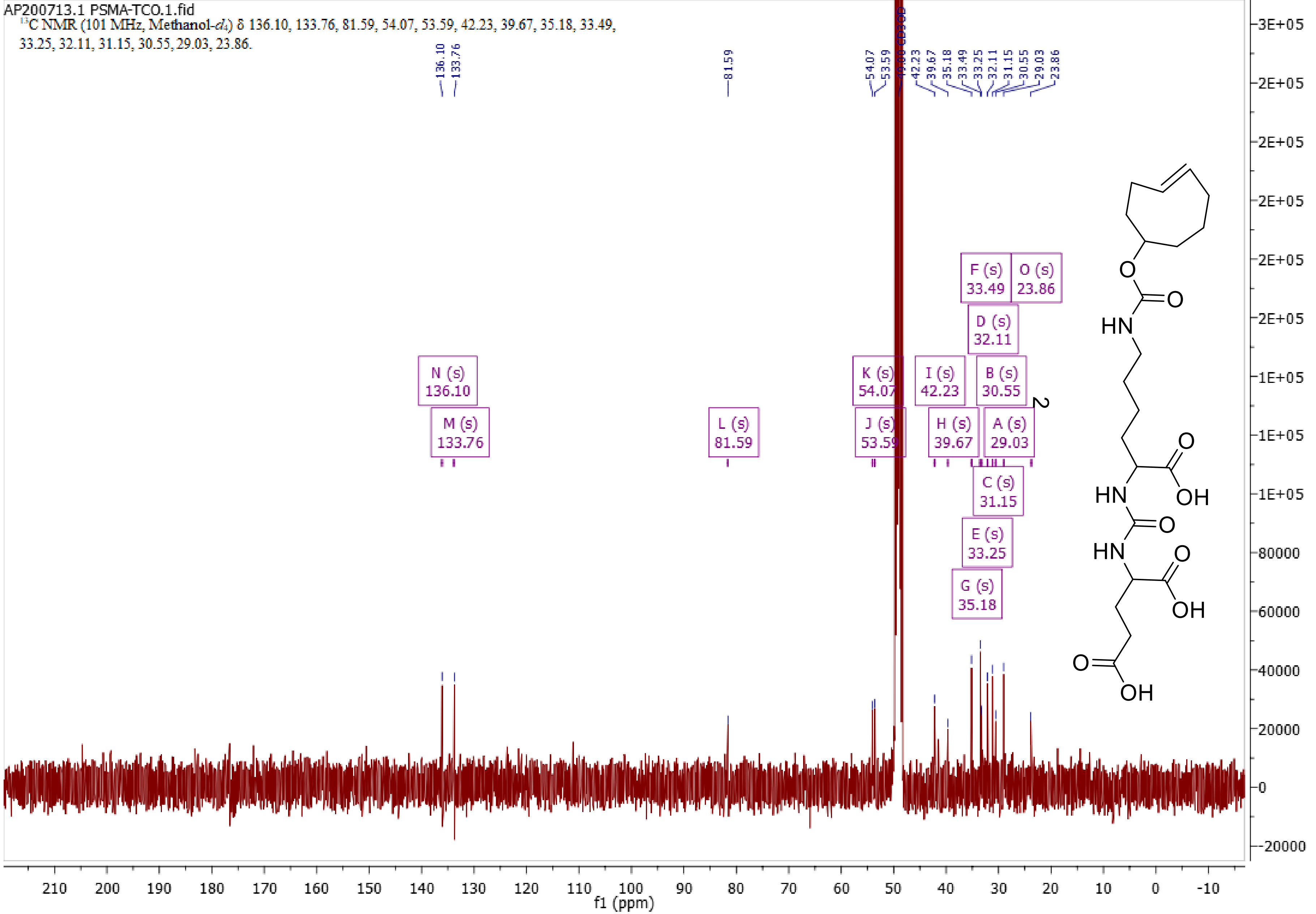

Supplementary Figure S2: C-NMR of PSMA-TCO (2) in d-Methanol

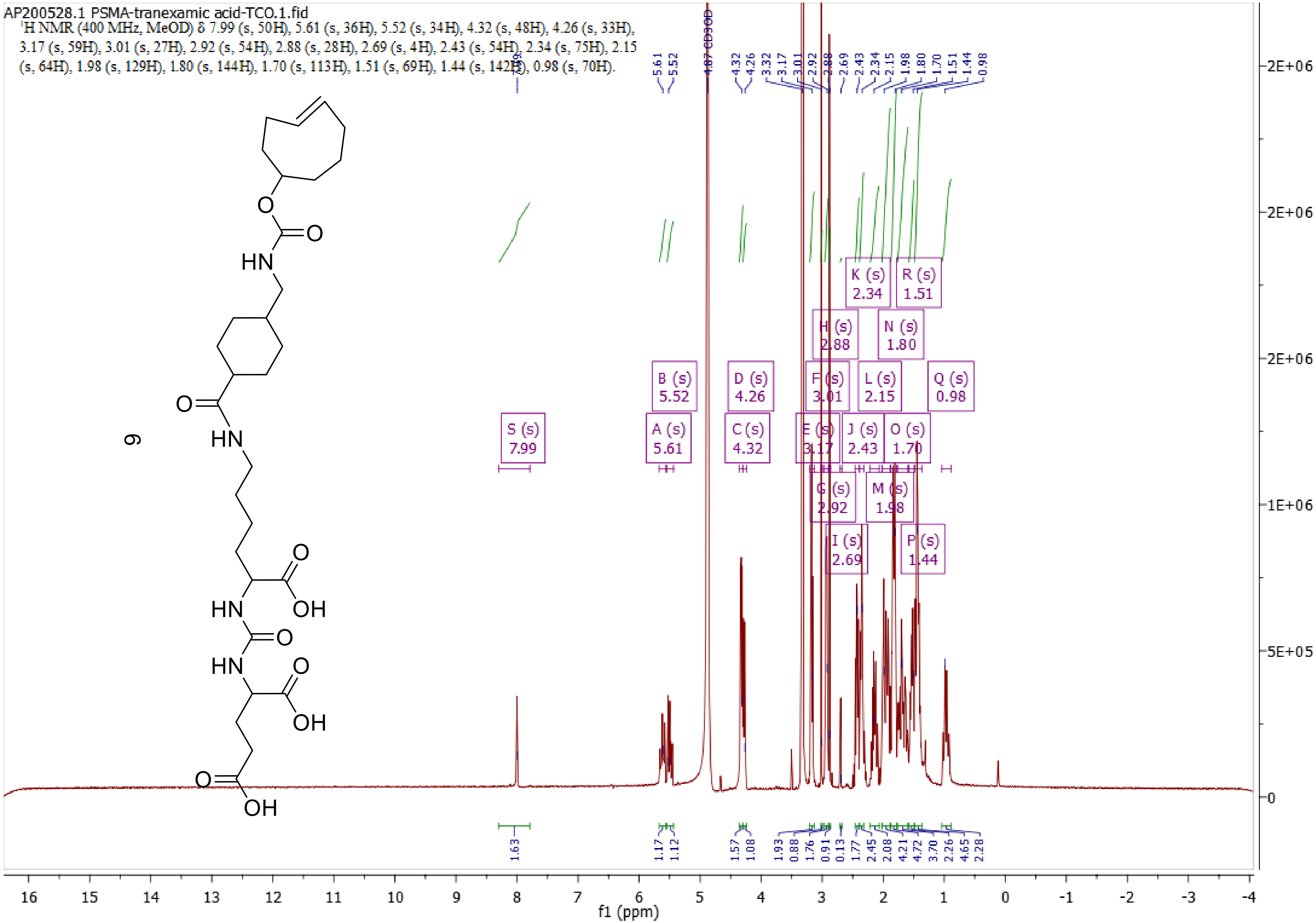

Supplementary Figure S3: H-NMR of PSMA-tranexamic acid-TCO (6) in d-Methanol

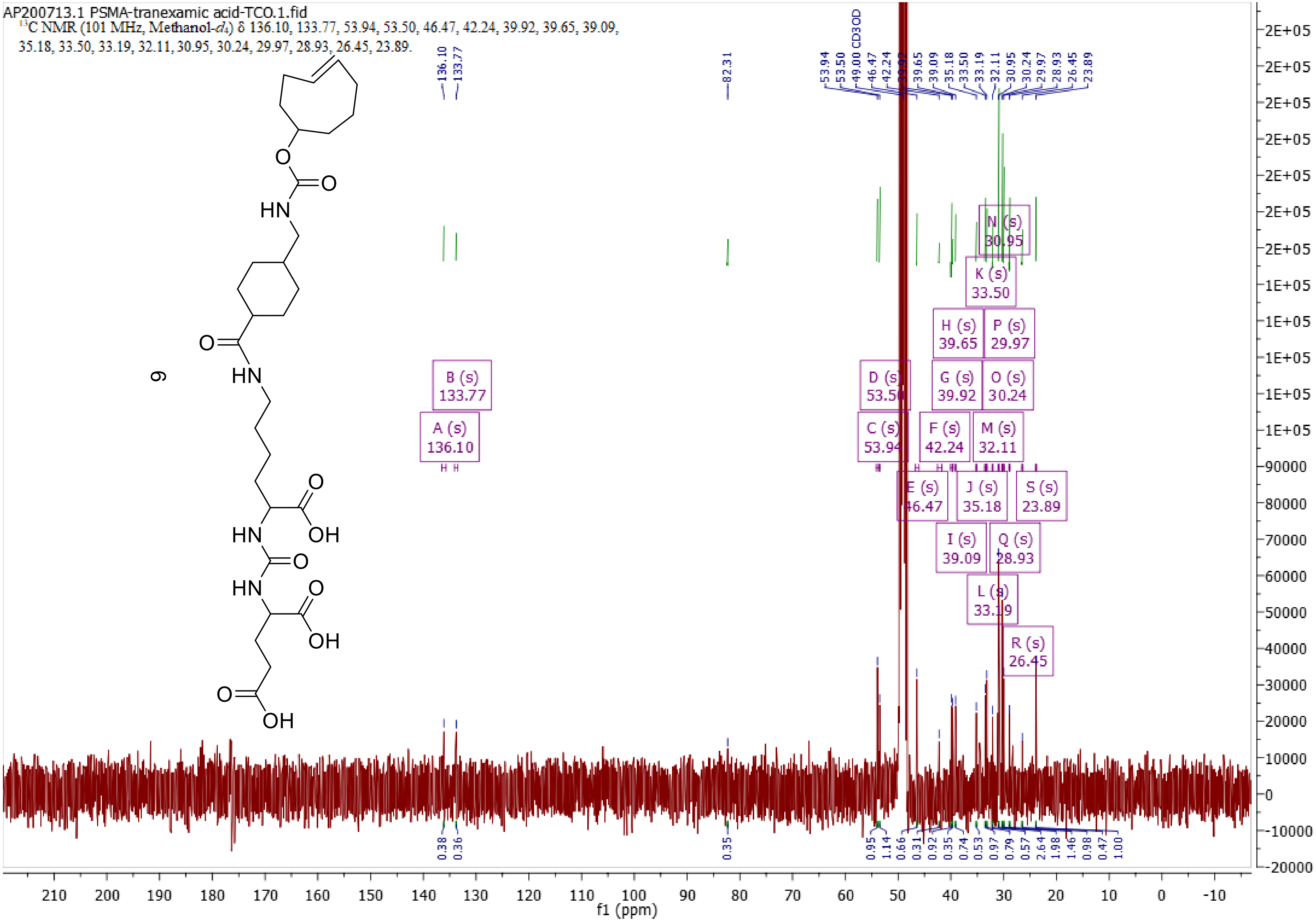

Supplementary Figure S4: 13C-NMR of PSMA-tranexamic acid-TCO (6) in d-Methanol

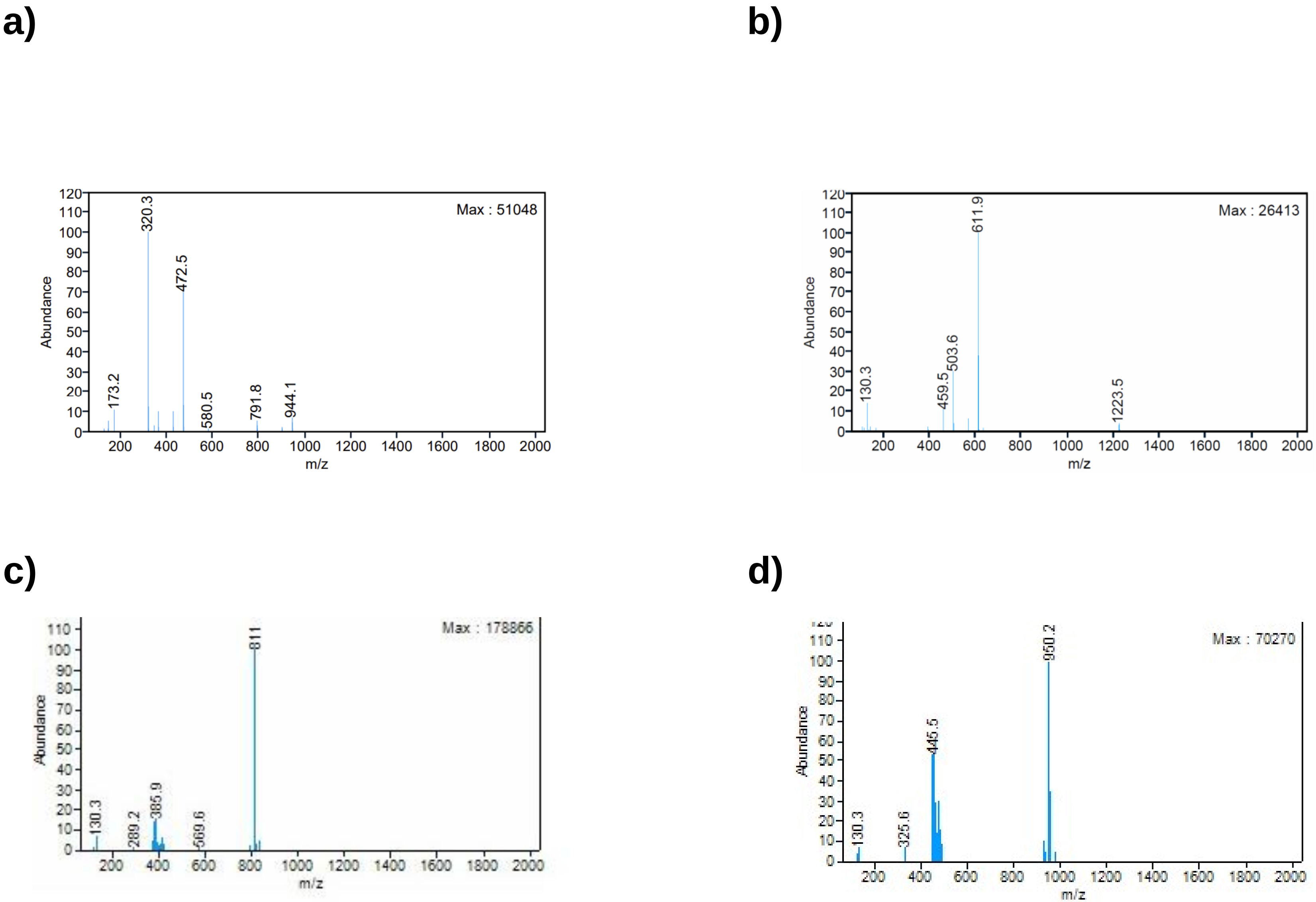

Supplementary Figure S5: **a)** LC-MS analysis of TCO-PSMA **b)** LC-MS analysis of TCO-tranexamic acid-PSMA **c)** LC-MS analysis of AM1-PSMA **d)** LC-MS analysis of AM1-tranexamic aicd-PSMA

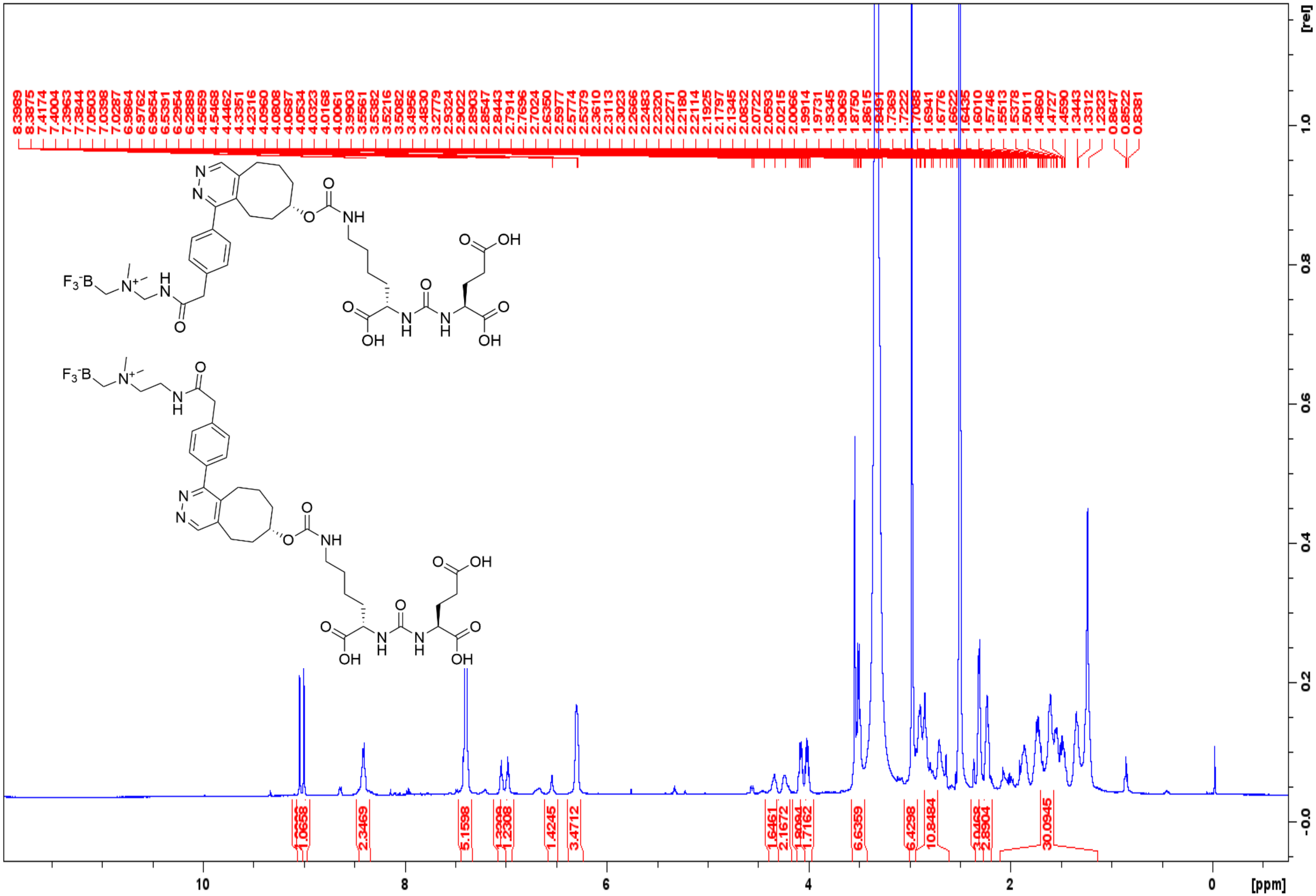

Supplementary Figure S6 : ^{1}H NMR of TzAmBF_3 ligated (2)

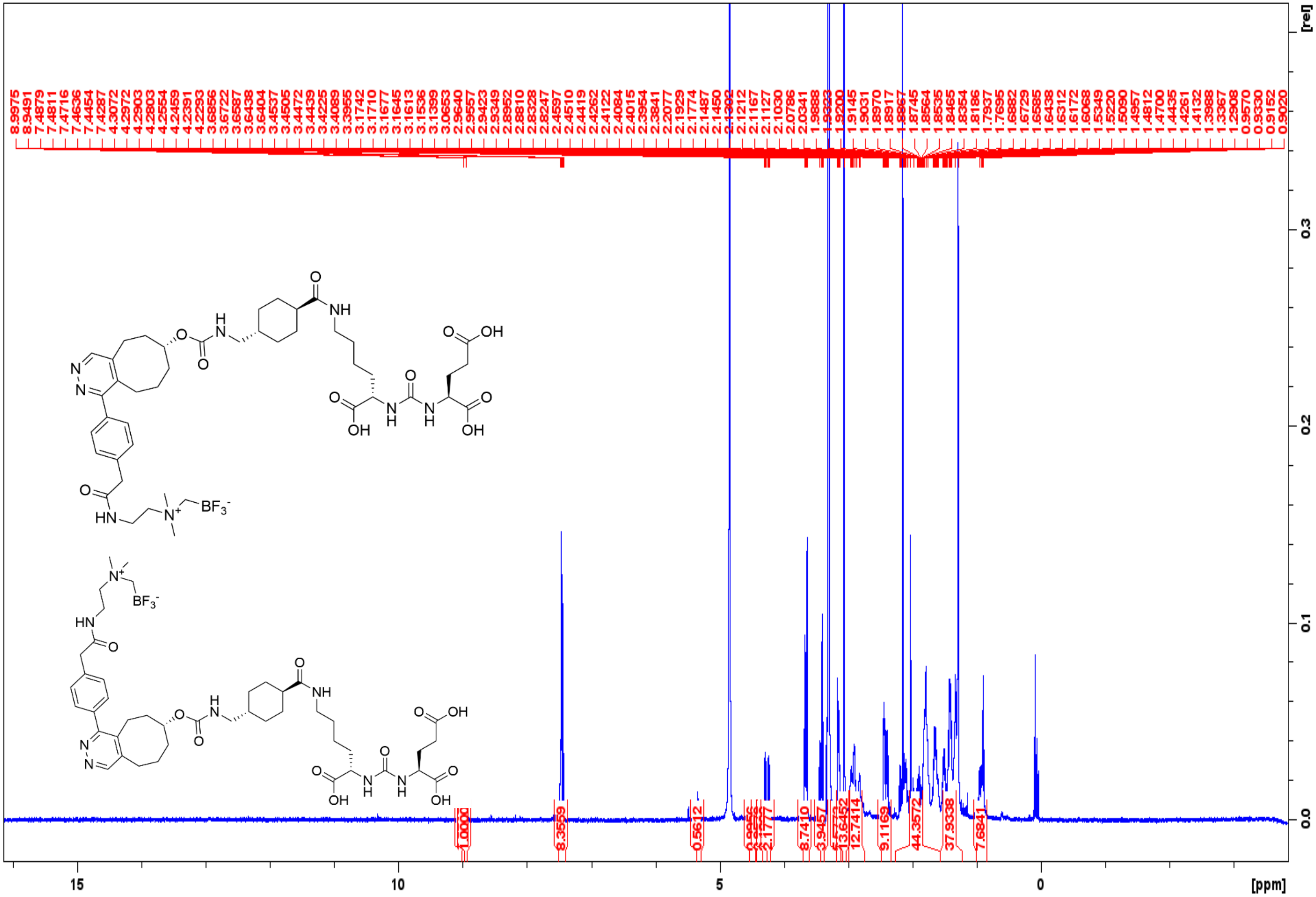

Supplementary Figure S7: ^{1}H NMR of $TzAmBF_3$ ligated (6)

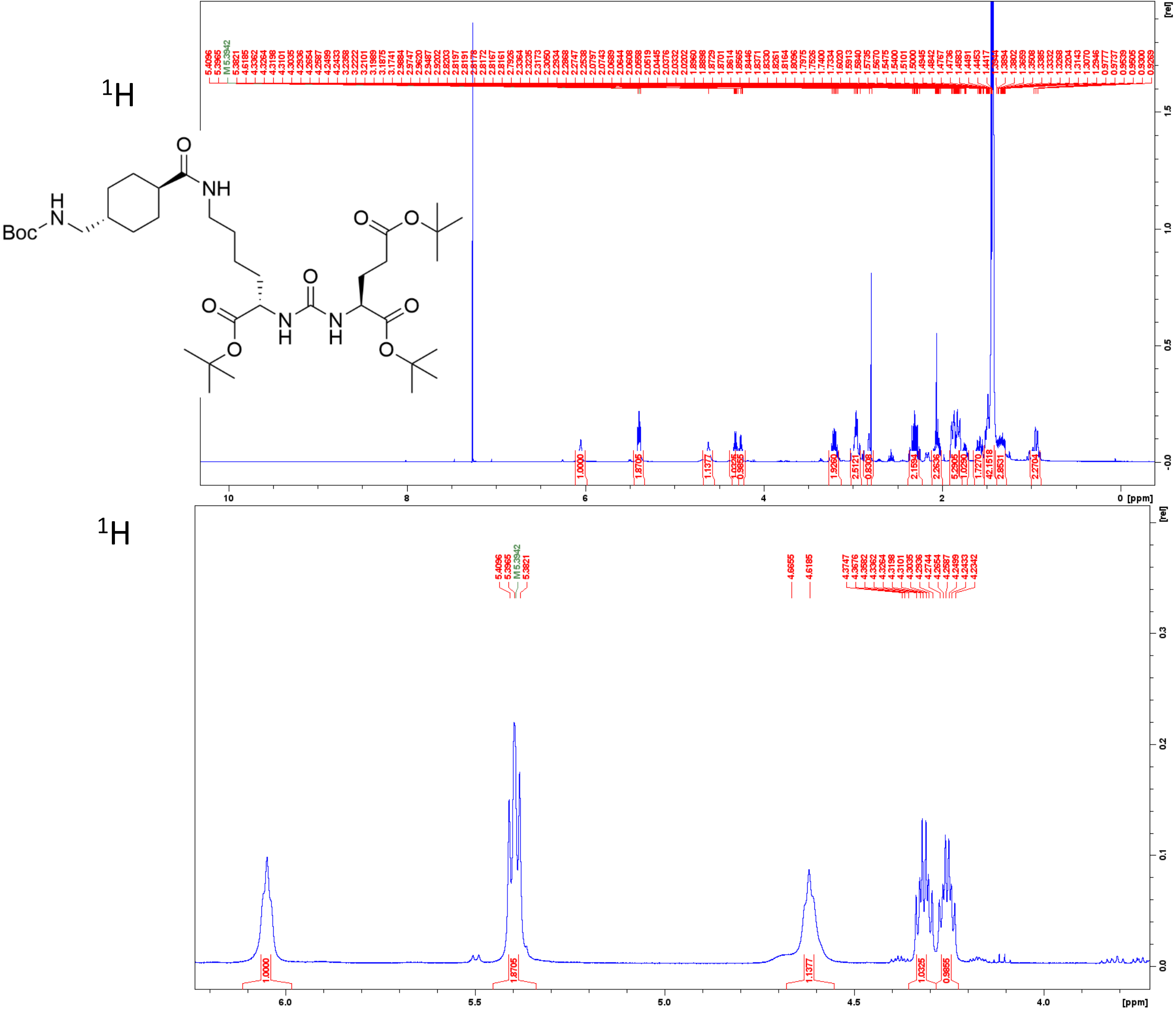

Supplementary Figure S8: ^{1}H NMR spectra of di-*tert*-butyl-(((S)-1-(*tert*-butoxy)-6-((1r,4S)-4-(((tert-butoxycarbonyl)amino)-methyl)cyclohexane-1-carboxamido)-1-oxohexan-2-yl)carbamoyl)-L-glutamate

^{1}H

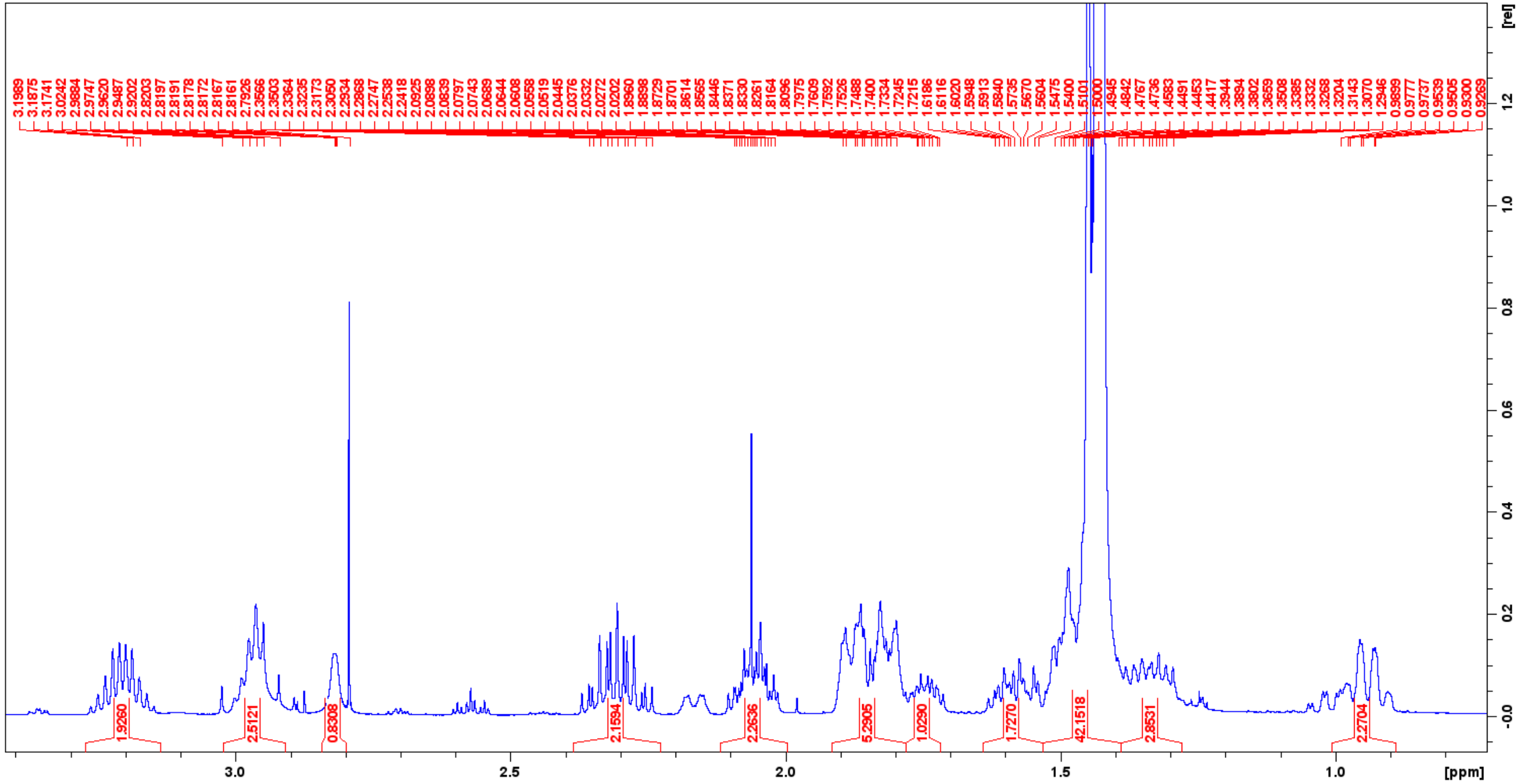

^{13}C

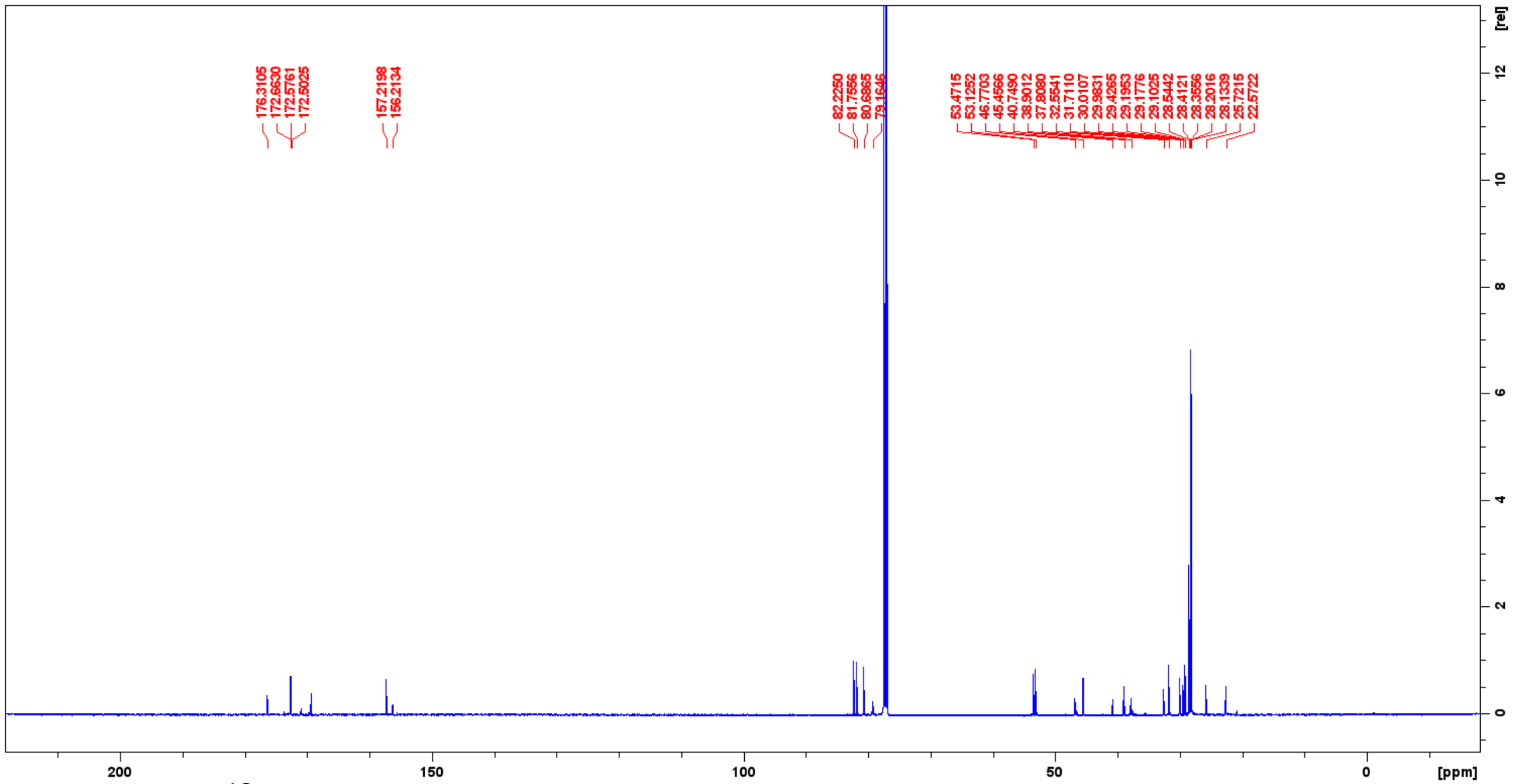

Supplementary Figure S9: ^{13}C NMR spectra of di-*tert*-butyl-(((S)-1-(*tert*-butoxy)-6-((1r,4S)-4-(((tert-butoxycarbonyl)amino)-methyl)cyclohexane-1-carboxamido)-1-oxohexan-2-yl)carbamoyl)-L-glutamate

^{13}C

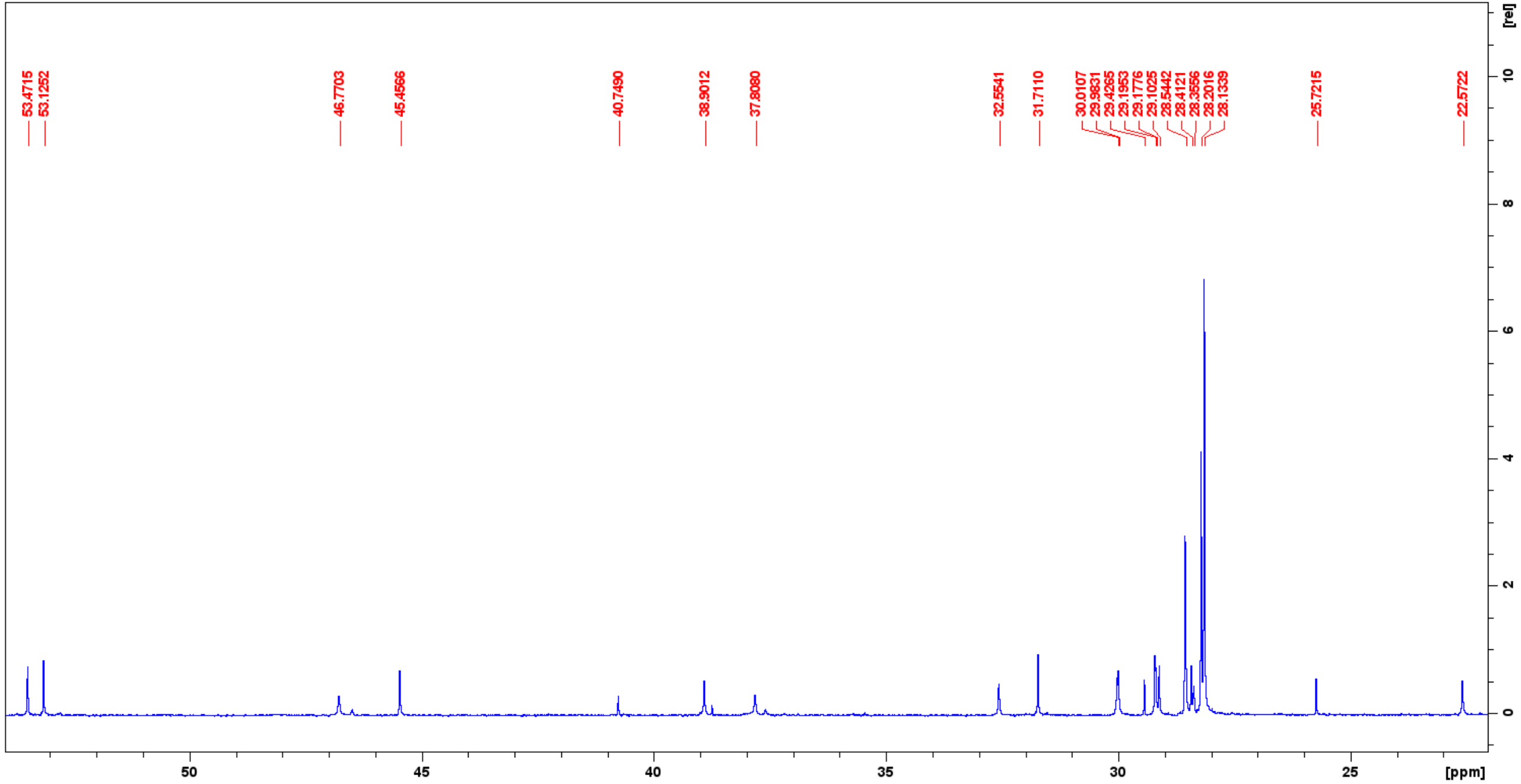

COSY

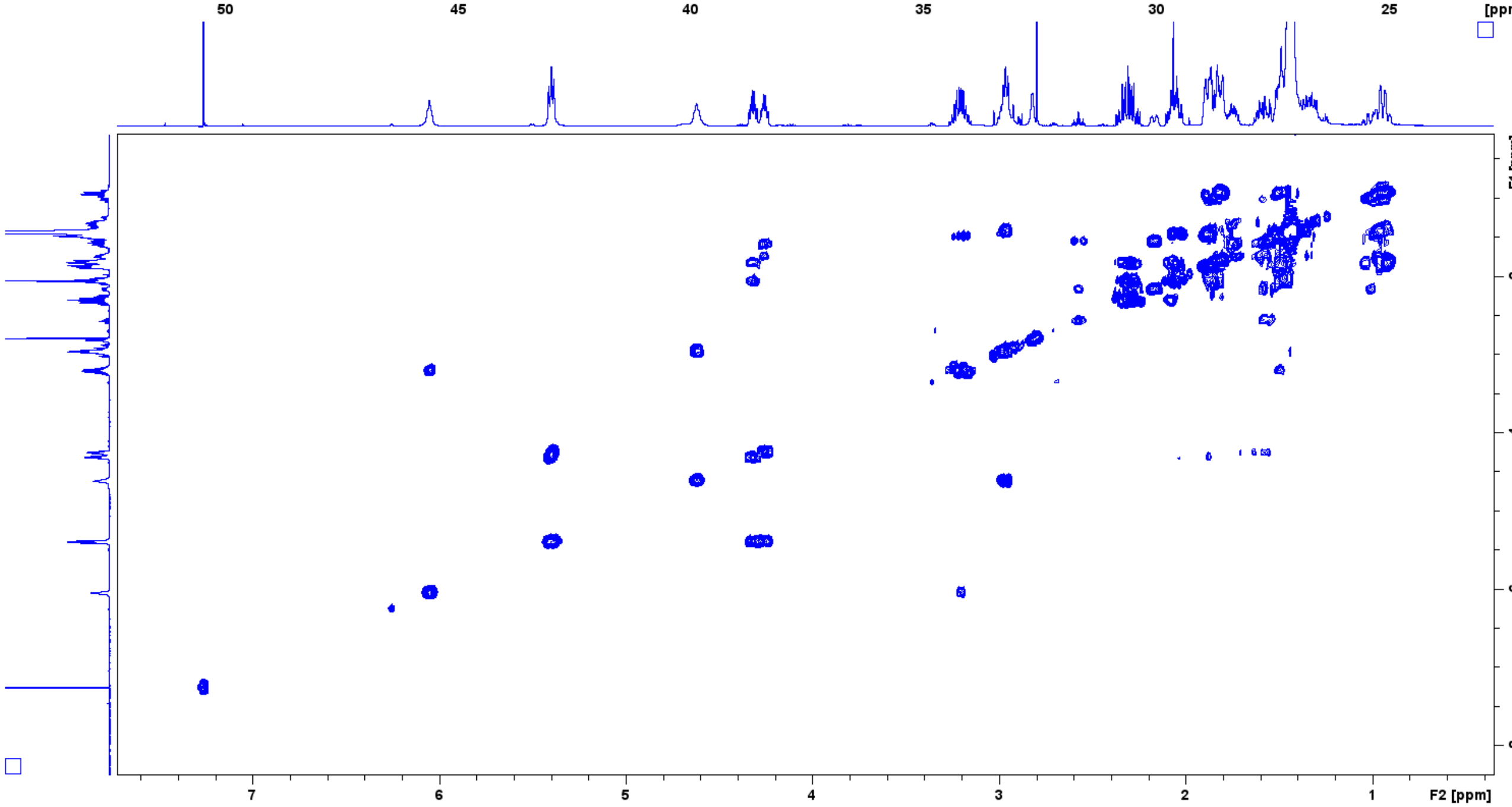

Supplementary Figure S10: COSY NMR spectra of di-*tert*-butyl-(((S)-1-(*tert*-butoxy)-6-((1r,4S)-4-(((tert-butoxycarbonyl)amino)-methyl)cyclohexane-1-carboxamido)-1-oxohexan-2-yl)carbamoyl)-L-glutamate

HSQC

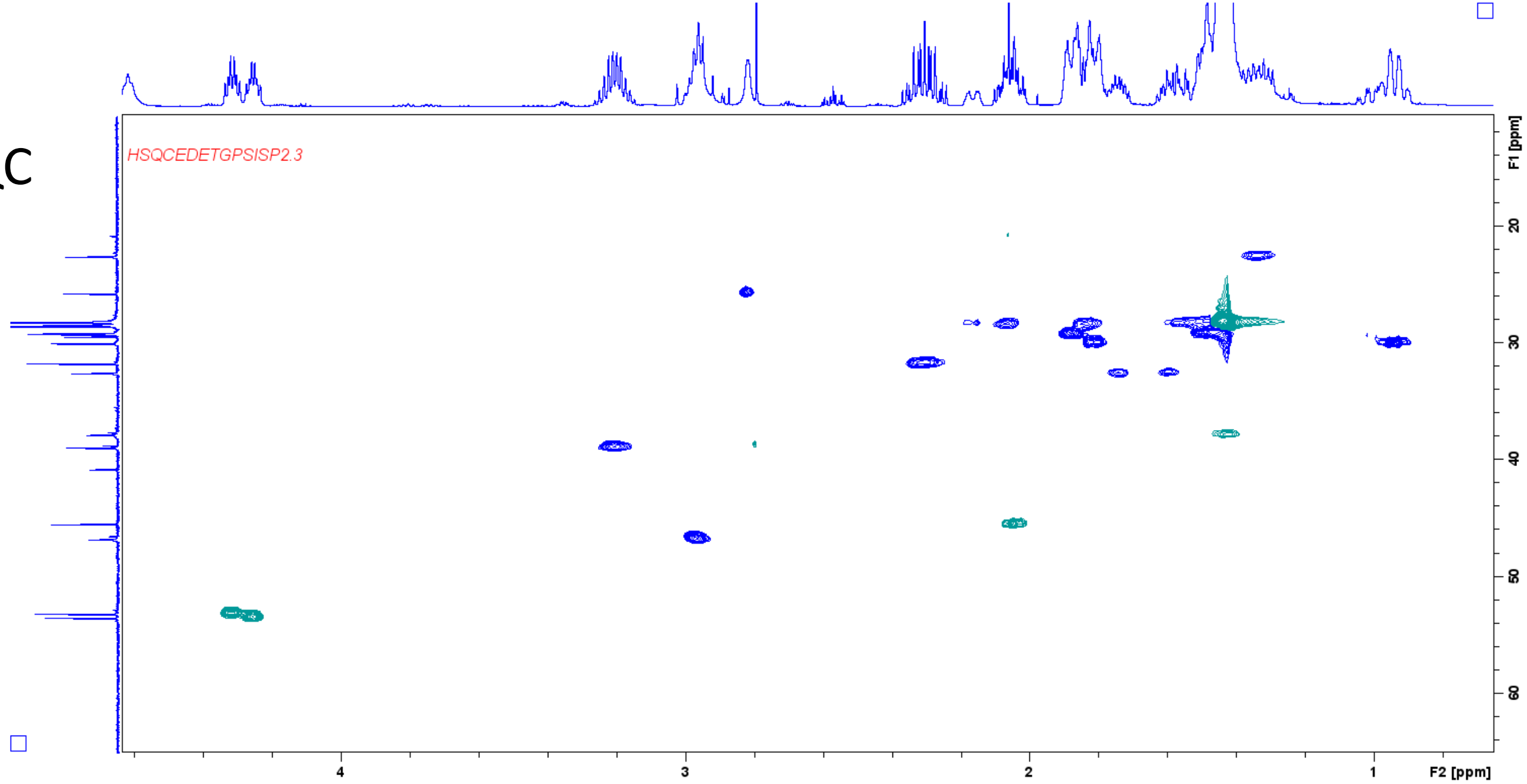

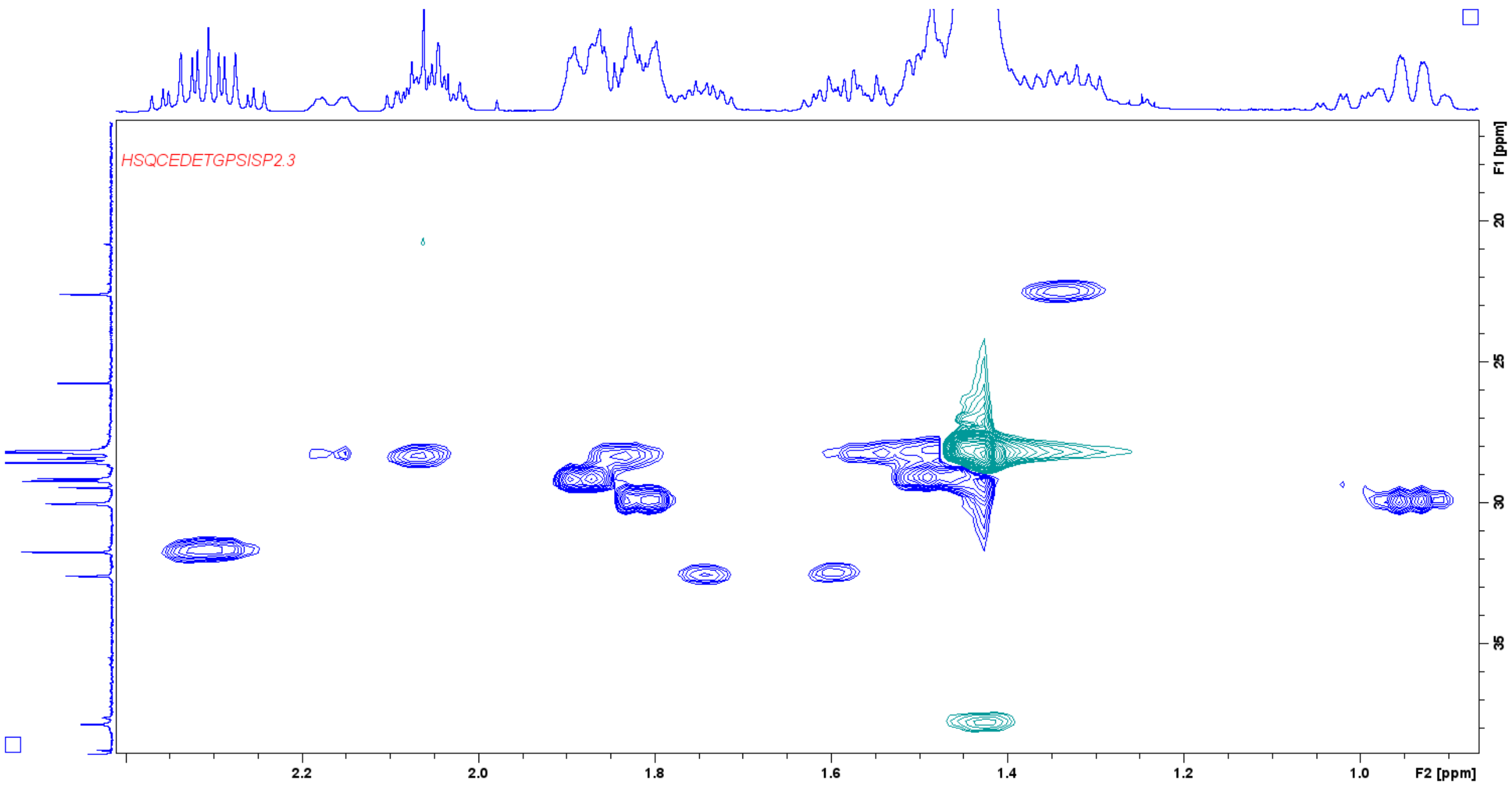

Supplementary Figure S11: HSQC NMR spectra of di-*tert*-butyl-(((S)-1-(*tert*-butoxy)-6-((1r,4S)-4-(((tert-butoxycarbonyl)amino)-methyl)cyclohexane-1-carboxamido)-1-oxohexan-2-yl)carbamoyl)-L-glutamate

HMBC

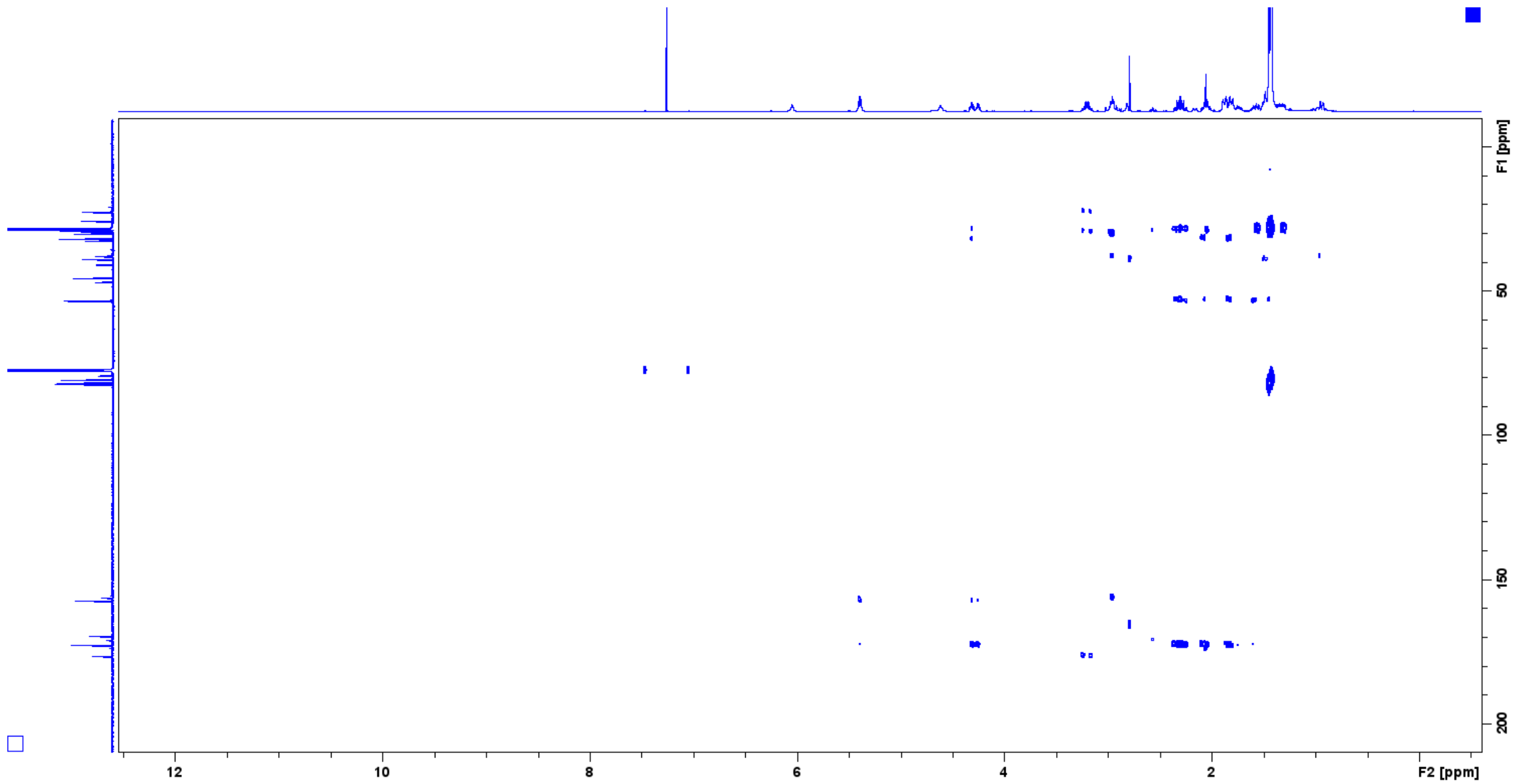

HMBC

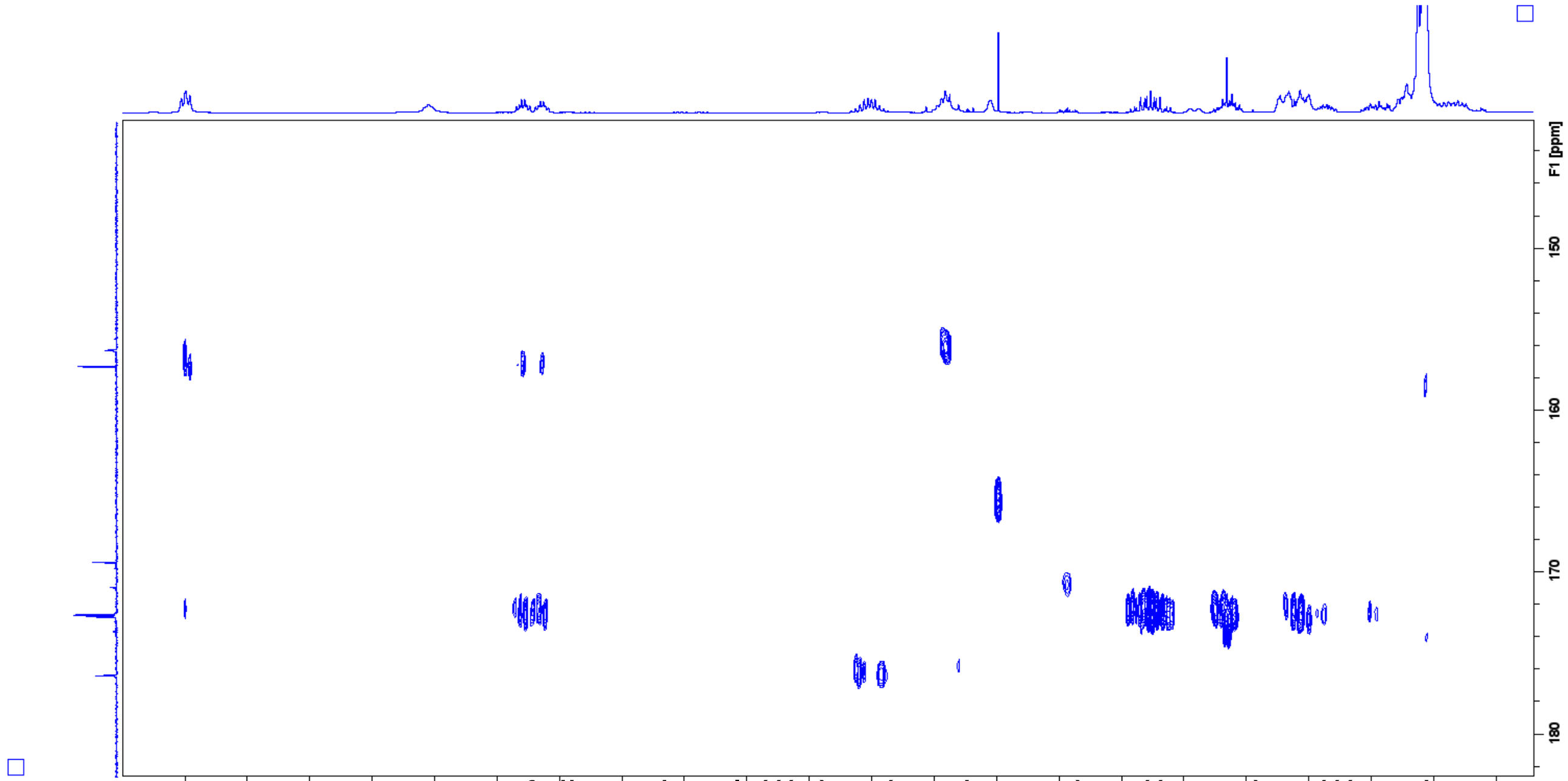

Supplementary Figure S12: HMBC NMR spectra of di-*tert*-butyl-(((S)-1-(*tert*-butoxy)-6-((1r,4S)-4-(((tert-butoxycarbonyl)amino)-methyl)cyclohexane-1-carboxamido)-1-oxohexan-2-yl)carbamoyl)-L-glutamate

HMBC

1D TOCSY

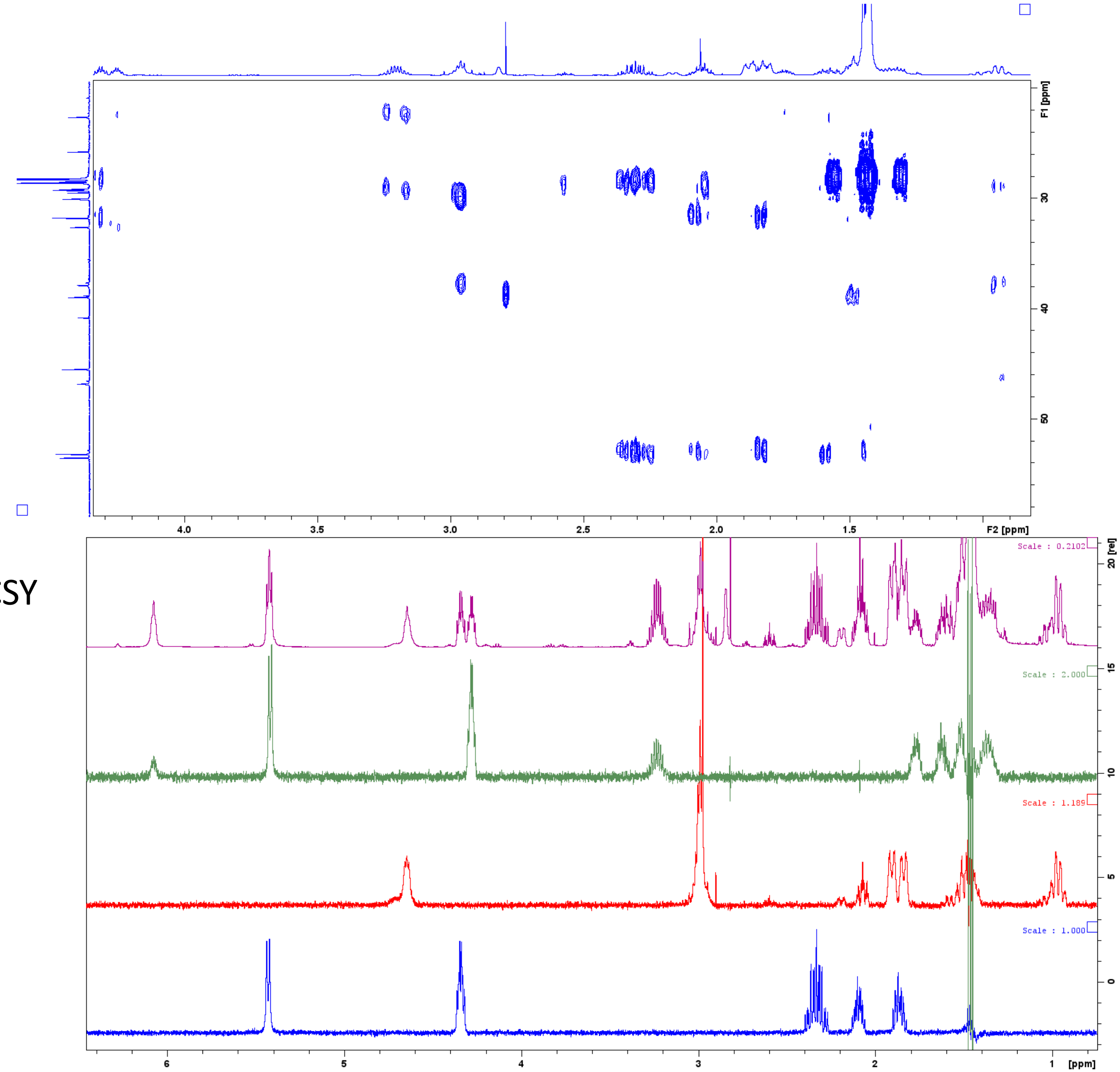

Supplementary Figure S13: 1D TOCSY NMR spectra of the spin systems in di-*tert*-butyl-(((S)-1-(*tert*-butoxy)-6-((1r,4S)-4-(((tert-butoxycarbonyl)amino)methyl)cyclohexane-1-carboxamido)-1-oxohexan-2-yl)carbamoyl)-L-glutamate

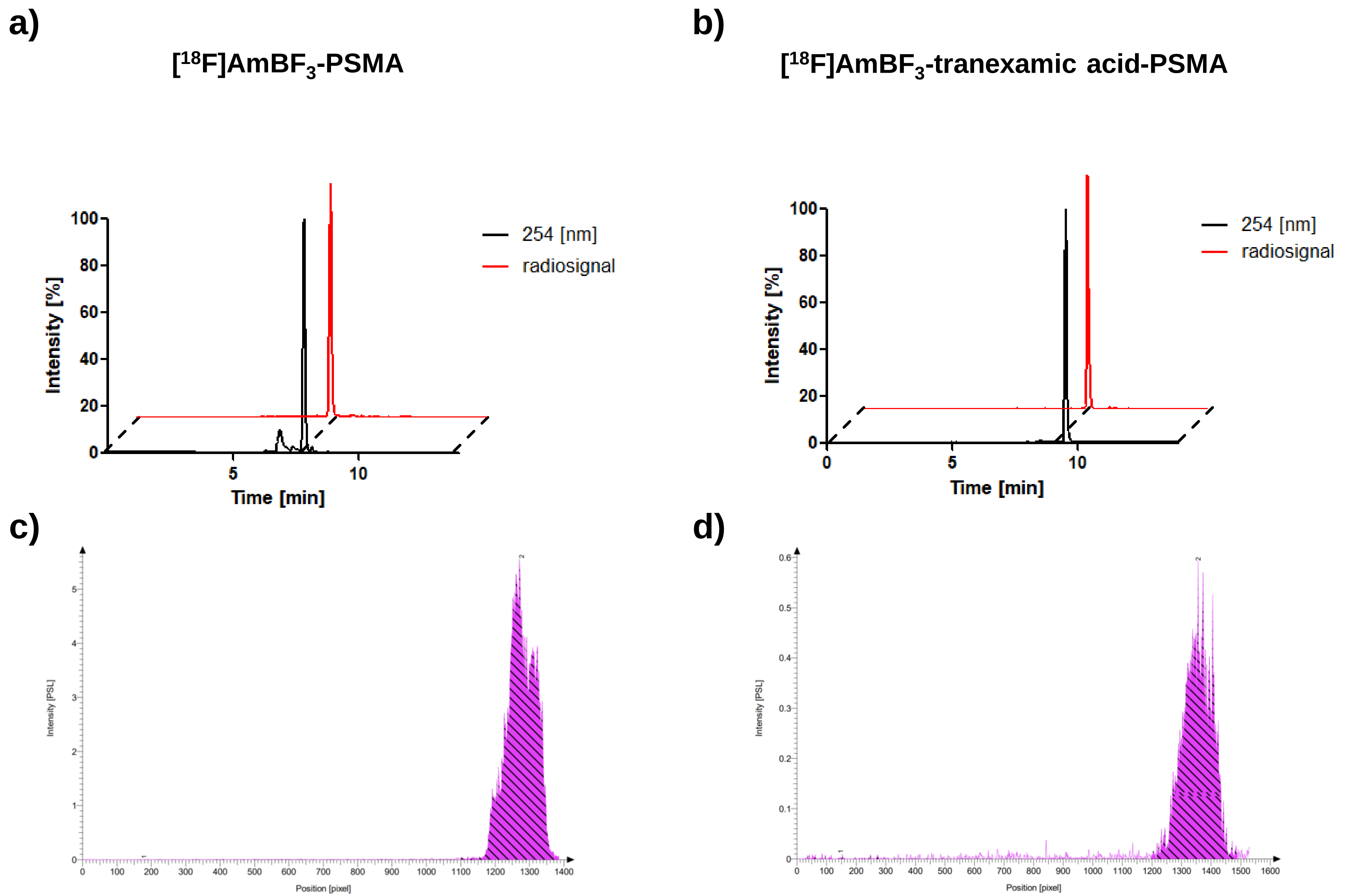

Supplementary Figure S14: **a)** HPLC chromatogram (UV- and radiosignal) of $[^{18}F]$ $AmBF_3$-PSMA **$[^{18}F]$7** **b)** HPLC chromatogram (UV- and radiosignal) of $[^{18}F]AmBF_3$-tranexamic acid-PSMA **$[^{18}F]$8** **c)** TLC analysis of $[^{18}F]$ $AmBF_3$-PSMA **$[^{18}F]$7** **d)** TLC analysis of $[^{18}F]AmBF_3$-tranexamic acid-PSMA **$[^{18}F]$8**

	[^{18}F] AmBF$_3$-PSMA			[^{18}F] AmBF$_3$-PSMA blocking			[^{18}F]AmBF$_3$-tranexamic acid-PSMA			[^{18}F]AmBF$_3$-tranexamic acid-PSMA blocking		
Blood	0,96	±	0,96	0,53	±	0,19	0,84	±	0,33	0,57	±	0,14
Spleen	21,21	±	12,12	2,15	±	1,12	37,89	±	8,55	2,32	±	0,48
Pancreas	0,29	±	0,09	0,37	±	0,25	0,55	±	0,19	0,50	±	0,40
Kidney	128,69	±	35,01	2,40	±	0,30	190,57	±	18,69	3,39	±	1,36
Gallbladder	30,80	±	4,67	32,68	±	28,48	27,83	±	8,68	15,55	±	19,94
Liver	0,63	±	0,29	0,51	±	0,13	0,57	±	0,05	0,48	±	0,04
Lung	0,88	±	0,23	0,54	±	0,31	1,68	±	0,09	0,65	±	0,14
Heart	0,41	±	0,18	0,30	±	0,11	0,89	±	0,22	0,39	±	0,05
Skeletal muscle	0,22	±	0,05	0,43	±	0,35	0,32	±	0,17	2,02	±	3,16
Bone (tibia)	2,22	±	0,79	8,50	±	4,27	2,41	±	0,65	4,08	±	1,26
Bone (occipital)	3,13	±	1,10	4,59	±	1,39	2,82	±	0,47	5,41	±	0,06
Brain	0,06	±	0,04	0,13	±	0,08	0,07	±	0,02	0,15	±	0,10
Stomach	0,48	±	0,17	1,18	±	0,88	0,42	±	0,09	0,24	±	0,07
Small intestines	11,47	±	4,20	6,71	±	6,42	5,95	±	2,39	1,73	±	1,22
Large intestines	1,38	±	0,89	5,15	±	7,92	0,32	±	0,10	2,14	±	3,15
Skin	0,59	±	0,42	1,43	±	1,85	0,70	±	0,21	0,74	±	0,45
Injection site	1,80	±	0,84	1,24	±	0,08	2,90	±	1,19	5,69	±	6,07
Tumor	7,52	±	0,70	1,19	±	0,20	12,87	±	4,83	1,90	±	0,66

Tumor/blood	7,86	2,26	15,25	3,32
Tumor/muscle	33,93	2,77	39,70	0,94

Supplementary Figure S15: Biodistribution results of **a)** [^{18}F]AM1-PSMA, **b)** [^{18}F]AM1-tranexamic acid-PSMA after 60 min (100 µL injected, 0.01 mM solution in PBS) in awake animals. Blocking was performed by injection of 2-PMPA (100 µL, 0.4 mM) 30 min prior the tracer injection

[^{18}F]AmBF$_3$-PSMA

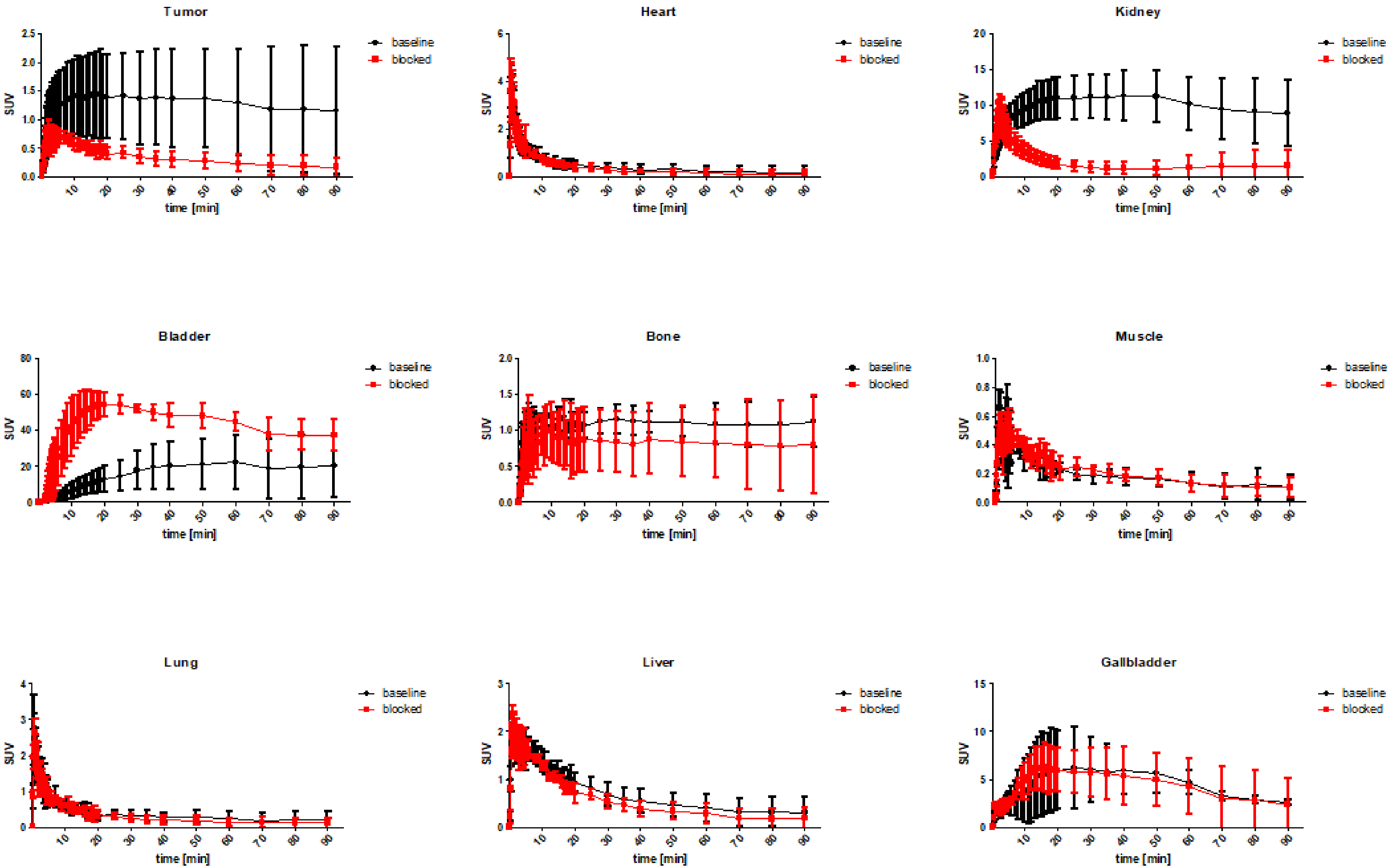

Supplementary Figure S16: SUV values for [^{18}F]AmBF$_3$-PSMA

[^{18}F]AmBF$_3$-tranexamic acid-PSMA

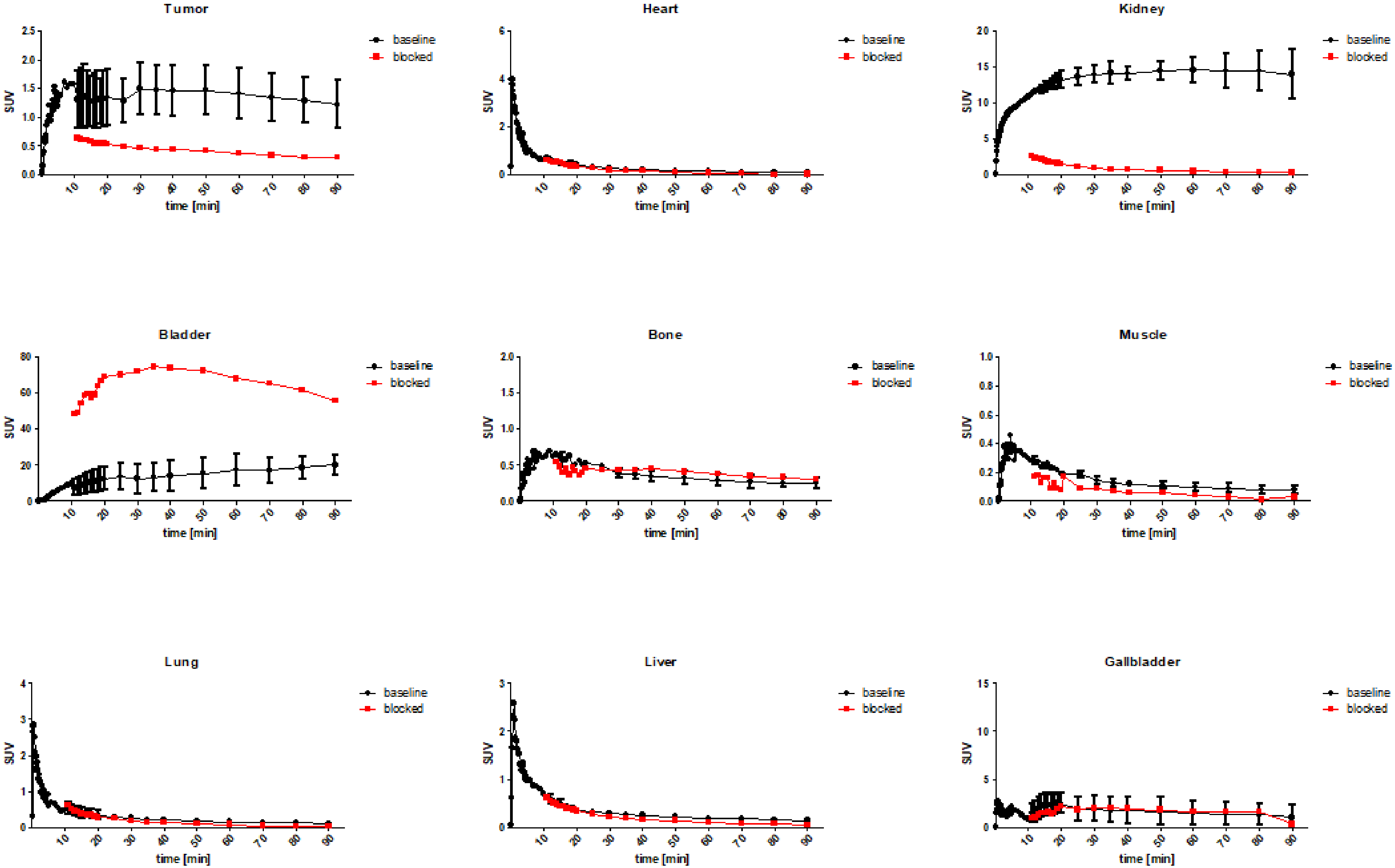

Supplementary Figure S17: SUV values for [^{18}F]AmBF$_3$-tranexamic acid-PSMA
